# *Mycoplasma hyopneumoniae* J elicits an antioxidant response and decreases the expression of ciliary genes in infected swine epithelial cells

**DOI:** 10.1038/s41598-020-70040-y

**Published:** 2020-08-13

**Authors:** Scheila G. Mucha, Mariana G. Ferrarini, Carol Moraga, Alex Di Genova, Laurent Guyon, Florence Tardy, Sophie Rome, Marie-France Sagot, Arnaldo Zaha

**Affiliations:** 1 Centro de Biotecnologia, Universidade Federal do Rio Grande do Sul, Porto Alegre, Brazil; 2 Université de Lyon, Université Lyon 1, CNRS, Laboratoire de Biométrie et Biologie Evolutive UMR 5558, 69622 Villeurbanne, France; 3 ERABLE, Inria, Lyon, France; 4 Université Grenoble Alpes, CEA, INSERM, IRIG, Biology of Cancer and Infection UMR_S 1036, 38000 Grenoble, France; 5 Université de Lyon, Anses, Laboratoire de Lyon, UMR Mycoplasmoses des Ruminants, Lyon, France; 6 Université de Lyon, VetAgro Sup, UMR Mycoplasmoses des Ruminants, Lyon, France; 7 CarMeN Laboratory, INSERM 1060/INRA 1397, Université de Lyon, Faculté de Médecine Lyon-Sud, 69310 Pierre-Bénite, France

**Keywords:** Gene expression, Bacteriology, Pathogens, miRNAs, Transcriptomics

## Abstract

*Mycoplasma hyopneumoniae* is the most costly pathogen for swine production. Although several studies have focused on the host-bacterium association, little is known about the changes in gene expression of swine cells upon infection. To improve our understanding of this interaction, we infected swine epithelial NPTr cells with *M. hyopneumoniae* strain J to identify differentially expressed mRNAs and miRNAs. The levels of 1,268 genes and 170 miRNAs were significantly modified post-infection. Up-regulated mRNAs were enriched in genes related to redox homeostasis and antioxidant defense, known to be regulated by the transcription factor NRF2 in related species. Down-regulated mRNAs were enriched in genes associated with cytoskeleton and ciliary functions. Bioinformatic analyses suggested a correlation between changes in miRNA and mRNA levels, since we detected down-regulation of miRNAs predicted to target antioxidant genes and up-regulation of miRNAs targeting ciliary and cytoskeleton genes. Interestingly, most down-regulated miRNAs were detected in exosome-like vesicles suggesting that *M. hyopneumoniae* infection induced a modification of the composition of NPTr-released vesicles. Taken together, our data indicate that *M. hyopneumoniae* elicits an antioxidant response induced by NRF2 in infected cells. In addition, we propose that ciliostasis caused by this pathogen is partially explained by the down-regulation of ciliary genes.

## Introduction

Respiratory diseases are among the major health problems in the pig farming industry. *Mycoplasma hyopneumoniae* is the causative agent of swine enzootic pneumonia, a chronic respiratory disease that affects herds worldwide. Although *M. hyopneumoniae* does not cause high mortality, it is considered the most expensive pathogen for swine production^1^. This is mainly due to the costs of treatment and vaccination and to losses related to decreased animal performance. In addition, *M. hyopneumoniae* is essential for the establishment of secondary pathogens in the host, which leads to a significant increase in mortality^2^. *M. hyopneumoniae* attaches to the cilia of the tracheal epithelial cells with participation of adhesins^3^, resulting in ciliostasis and cell death^4^. Besides adhesins, virulence factors are not well understood in this bacterium. Nevertheless, a recent study from our group indicated hydrogen peroxide production from glycerol and myo-inositol metabolism as important traits that might be related with pathogenesis and with the predominance of *M. hyopneumoniae* in the swine respiratory tract^5^.

MicroRNAs (miRNAs) belong to a class of small non-coding RNAs (ncRNAs) of 18–24 nucleotides (nt) in part responsible for post-transcriptional gene regulation in eukaryotes. These evolutionarily conserved molecules influence fundamental biological processes, including cell proliferation, differentiation, apoptosis, immune response, and metabolism^6, 7^. The binding of miRNAs to target mRNAs changes the mRNA stability and translation efficiency^8^, leading to degradation, suppression or up-regulation of the target mRNAs^6, 9^. Interactions between miRNA and mRNA are complex; one single miRNA can target a large number of genes belonging to diverse functional groups. Alternatively, the 3’-UTR of a single mRNA can be targeted by multiple miRNAs^10, 11^. By modulating miRNA abundance, it is thus possible to fine-tune the expression of proteins within the cell in a very precise manner^6, 11^.

Recently, it was widely reported that miRNAs can be packed into exosomes and transferred to neighboring or distant cells to regulate cell function^7, 12–14^. Exosomes are small membrane vesicles (50–150 nm) released from eukaryotic cells both constitutively and upon induction, under normal and pathological conditions^13, 15^. These vesicles are involved in several cellular functions and have the potential to selectively interact with specific target cells^16, 17^. In addition to miRNAs, exosomes can transmit information among cells by transferring proteins, lipids and nucleic acids that seem to be selected non-randomly, with some specific populations of molecules being preferentially packaged into the vesicles^13, 15^. As an efficient cellular signaling and communication system, the release of exosomes by infected host cells has been recognized as a common phenomenon, in some cases beneficial to the host and in others beneficial to the pathogen^18^.

Host-pathogen interactions result in signaling and physiological modifications in the host cells that induce differential miRNA expression and miRNA-mediated post-transcriptional regulation of genes involved in immune response and several other cellular pathways^19, 20^. Therefore, simultaneous identification of differentially expressed miRNAs and mRNAs provides a comprehensive view on host-pathogen interactions during the infection and the disease establishment process. In recent years, efforts have been made to identify miRNAs regulated by infection in several mammalian hosts^8, 20–22^. However, the identification of miRNAs during infection of swine cells with *M. hyopneumoniae* has not been investigated so far. To improve our understanding on the *M. hyopneumoniae*-host interaction, we sequenced and analyzed both the mRNAs and miRNAs of a swine tracheal epithelial cell line infected with *M. hyopneumoniae* strain J. In addition, we identified miRNAs differentially expressed (DE) in the extracellular milieu and in exosome-like vesicles released by the infected cells, which play an important role in cell-cell communication and in the dissemination of host and pathogen-derived molecules during infection^15^. The simultaneous identification of miRNAs and mRNAs will help us draw a full picture of the changes in gene expression and the possible regulatory mechanisms of host cells during the disease establishment.

## Results and discussion

### *M. hyopneumoniae* strain J adhered to NPTr cells

To analyze the differential expression of New-born Pig Trachea (NPTr) cells during the infection with *M. hyopneumoniae*, we first observed the infection by immunofluorescence microscopy. These analyses were performed to detect the adherence of *M. hyopneumoniae* strain J to NPTr cells. Supplementary Figure S1 shows the co-localization of *M. hyopneumoniae* with NPTr cells, corroborating the success of the infection. As few bacteria adhered to the cells within a short period of time (1 vs. 24 h), we chose to analyze the transcriptional alterations of NPTr cells at 24 h post-infection. *M. hyopneumoniae* strain J was chosen because previous infection assays showed that highly virulent strains, such as 7448 or 7422, damaged the host cells and we were not able to recover RNA with good sequencing quality. Although this strain is considered attenuated and incapable of causing disease in vivo^23, 24^, our results show that *M. hyopneumoniae* is capable of adhering to the swine epithelial cells, as previously reported by Burnett et al.^25^.

### mRNA expression profiles

A total of 6 mRNA libraries were generated with the Illumina HiSeq2500 platform. A summary of the samples is provided in Table 1 and a diagram to explain the experimental design is given in Supplementary Fig. S2. The raw reads were submitted to the NCBI Sequence Read Archive under accession number PRJNA545822. After removing adapters and filtering low quality reads, mRNA-seq yielded around 40 million paired-end reads in all 6 samples (approx. 97% of raw reads). Trimming and mapping information for each mRNA sample is available in Supplementary Table S1. Around 85% of the filtered reads were mapped against the porcine genome (Sscrofa10.2—Ensembl release 89) and 40% against annotated genes (Supplementary Table S1).

**Table 1:** Samples detailed information. The experimental design with the description of the samples is also explained in Fig. S2.

Sample ID	Sample name	RNA type	Compartment		Condition	Replicates
mRNA 1-3	mRNA CTRL	mRNA	Intracellular		Control	3
mRNA 4-6	mRNA INF				Infected with MHP	
sRNA 1-3	sRNA INTRA CTRL	sRNA	Intracellular		Control	
sRNA 4-6	sRNA INTRA INF				Infected with MHP	
sRNA 7-8	sRNA EXTRA CTRL		Extracellular	Total extracellular sRNA	Control	2
sRNA 9-10	sRNA EXTRA INF				Infected with MHP	
sRNA 11	sRNA EXO CTRL			sRNA from exosome-like vesicles	Control	Pool (50 biological replicates)
sRNA 12	sRNA EXO INF				Infected with MHP	
sRNA 13	sRNA SN CTRL			sRNA from vesicle-free supernatant	Control	
sRNA 14	sRNA SN INF				Infected with MHP	

In the present study, we detected a total of 20,274 (out of 23,215) genes expressed with at least 10 counts across the 6 mRNA libraries from NPTr cells (Supplementary Table S2). Since we used an attenuated strain of *M. hyopneumoniae* to infect the swine cells, we did not expect extreme changes in gene expression. Indeed, most of the significant log2 of Fold Changes (LogFC) of the DE genes analyzed in our results were below 1 (for up-regulated genes) or above -1 (for down-regulated genes) (Supplementary Table S3). Nevertheless, even if the strain used (J) is considered attenuated, it generated a considerable immune response. A similar situation occurs in vaccine development, in which attenuated strains are used to generate immune response without causing lethal effects. Thus, we believe that infections with highly virulent strains of *M. hyopneumoniae* would be able to generate a more pronounced effect on gene expression than what was observed here. Regardless, our results provide a general overview of *M. hyopneumoniae* infection in the host cells.

**Figure 1:**
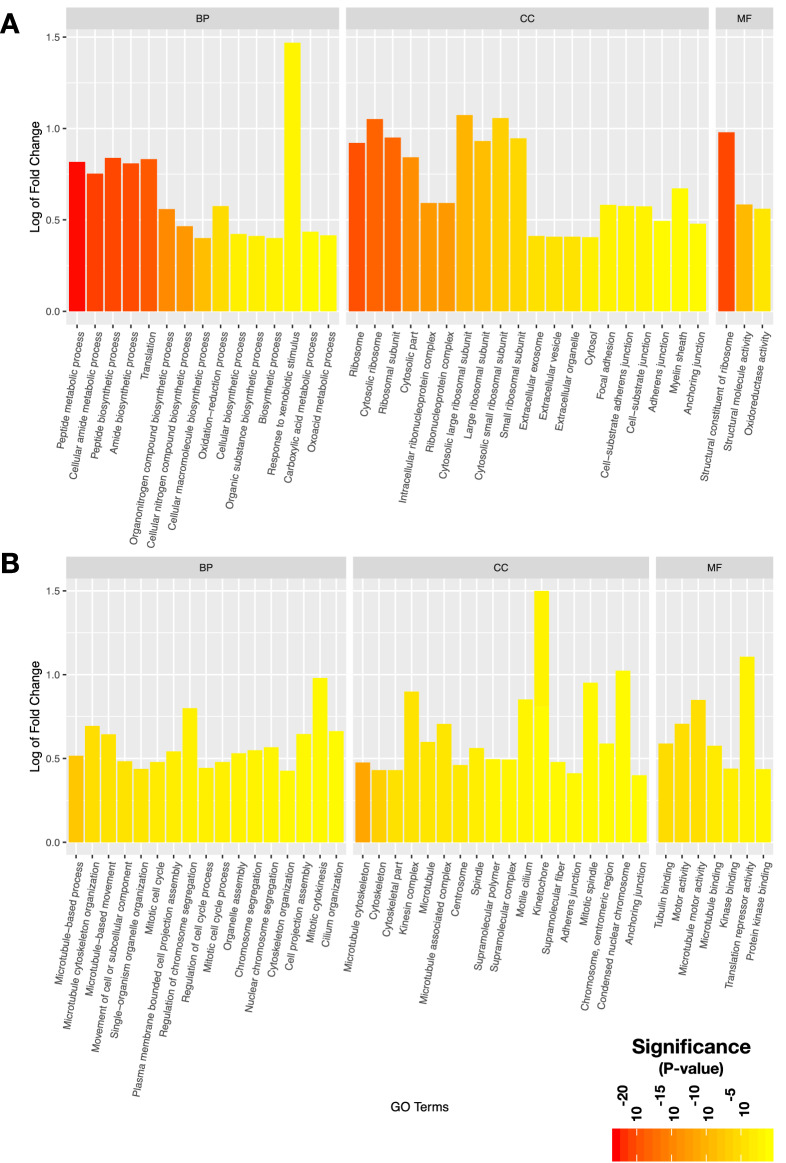
Significantly enriched Gene Ontology functions of DE mRNAs. **A**. Up-regulated genes were enriched in terms related to protein synthesis, oxidation-reduction activity and cell-cell communication (such as exosomes, anchoring and focal adhesion), either in biological process (BP), molecular function (MF) or cellular component (CC). **B.** Down-regulated genes showed an enrichment of terms related to cytoskeleton, ciliary function, cell cycle and cell division in all the three categories of GO.

In total, we detected 1,268 DE genes (p-adj $< 0.05$, 517 up-regulated and 751 down-regulated), from which 502 were common to two well known methods for the detections of DE genes, 721 were exclusive to DESeq2 and 45 were exclusive to EdgeR. Information from the top 15 up-regulated and down-regulated DE genes is provided in Table 2. The complete DE gene results are found in Supplementary Table S3. The results of gene ontology (GO) enrichment analysis for up-regulated and down-regulated genes in DESeq2 are shown separately in Fig. 1 (p-adj $< 0.05$ and absolute fold enrichment $\ge$ 0.1) and the complete results are found in Supplementary Table S4. For the up-regulated genes, the enriched terms either in biological process (BP), molecular function (MF) or cellular component (CC) were related to protein synthesis (translation/ribosome), oxidation reduction activity and cell-cell communication (such as exosomes, anchoring and focal adhesion functions) (Fig. 1A). For the down-regulated genes, the majority of the overrepresented terms in all three GO categories were related to cell cycle, cell division, cilia and cytoskeleton (Fig. 1B).

**Table 2:** Selected up- and down-regulated genes. Information about the top 15 up- and 15 down-regulated DE genes from both the DeSeq2 and EdgeR methods. Ordered by LogFC calculated by EdgeR.

DE genes			DESeq2		EdgeR		UP/DOWN
Gene ID	Associated gene name	Description	LogFC	P-adj	LogFC	P-adj	
ENSSSCG00000028871	*LOC396866*	Cystatin-A1	0.691	4,35E$-$24 *	5.375	1.02E$-$14	Upregulated
ENSSSCG00000030385	*C3*	Complement C3	1.427	4.69E$-$63	2.698	2.60E$-$43	Upregulated
ENSSSCG00000021728	*LGALS2*	Galectin 2	0.646	5,38E$-$15 *	1.507	3.71E$-$05	Upregulated
ENSSSCG00000028525	*SAA3*	Serum Amyloid A3	0.841	4.67E$-$25	1.211	2.88E$-$21	Upregulated
ENSSSCG00000001101	*SCGN*	Secretagogin, EF-Hand Calcium Binding Protein	0.554	6.74E$-$09	1.018	9.06E$-$05	Upregulated
ENSSSCG00000028982	*AKR1C4*	Aldo-Keto Reductase Family 1 Member C4	0.869	2.61E$-$76	0.953	4.61E$-$54	Upregulated
ENSSSCG00000025277	*AKR1CL1*	Aldo-Keto Reductase Family 1, Member C-Like 1	0.866	3.96E$-$76	0.949	5.46E$-$54	Upregulated
ENSSSCG00000004678	*DUOX2*	Dual Oxidase 2	0.515	1.99E$-$09	0.721	6.93E$-$09	Upregulated
ENSSSCG00000010146	*LGALS8*	Galectin 8	0.453	1.41E$-$06	0.691	2.39E$-$15	Upregulated
ENSSSCG00000025273	*CYP11A1*	Cytochrome P450 Family 11 Subfamily A Member 1	0.51	1.39E$-$11	0.648	3.80E$-$11	Upregulated
ENSSSCG00000003402	*PGD*	Phosphogluconate Dehydrogenase	0.611	3.62E$-$61	0.647	2.71E$-$36	Upregulated
ENSSSCG00000008959	*CXCL2*	C-X-C Motif Chemokine Ligand 2	0.491	5.15E$-$13	0.59	1.10E$-$11	Upregulated
ENSSSCG00000007435	*PLTP*	Phospholipid Transfer Protein	0.436	7.49E$-$09	0.544	3.54E$-$08	Upregulated
ENSSSCG00000010853	*EPHX1*	Epoxide Hydrolase 1	0.444	5.20E$-$15	0.503	1.76E$-$06	Upregulated
ENSSSCG00000000843	*TXNRD1*	Thioredoxin Reductase 1	0.426	7.45E$-$14	0.482	7.33E$-$13	Upregulated
ENSSSCG00000017135	*ZNF750*	Zinc Finger Protein 750	$-$0.741	7,84E$-$26 *	$-$4.899	3.43E$-$15	Downregulated
ENSSSCG00000009399	*CYSLTR2*	Cysteinyl Leukotriene Receptor 2	$-$0.439	1,01E$-$07 *	$-$1.034	2.57E$-$02	Downregulated
ENSSSCG00000013401	*DKK3*	Dickkopf WNT Signaling Pathway Inhibitor 3	$-$0.581	5.15E$-$13	$-$0.774	1.35E$-$12	Downregulated
ENSSSCG00000016273	*HTR2B*	5-Hydroxytryptamine Receptor 2B	$-$0.434	1.37E$-$05	$-$0.735	2.12E$-$03	Downregulated
ENSSSCG00000014232	*LOX*	Lysyl Oxidase	$-$0.464	1.06E$-$06	$-$0.718	1.39E$-$04	Downregulated
ENSSSCG00000011217	*NEK10*	NIMA Related Kinase 10	$-$0.418	2.80E$-$05	$-$0.689	4.20E$-$03	Downregulated
ENSSSCG00000003810	*UBE2U*	Ubiquitin Conjugating Enzyme E2 U (Putative)	$-$0.392	1.38E$-$04	$-$0.676	8.30E$-$03	Downregulated
ENSSSCG00000011441	*TNNC1*	Troponin C1, Slow Skeletal And Cardiac Type	$-$0.399	5.92E$-$05	$-$0.633	3.27E$-$03	Downregulated
ENSSSCG00000015413	*FGL2*	Fibrinogen Like 2	$-$0.487	1.81E$-$15	$-$0.554	1.64E$-$14	Downregulated
ENSSSCG00000008334	*MXD1*	MAX Dimerization Protein 1	$-$0.385	3.21E$-$06	$-$0.486	3.02E$-$05	Downregulated
ENSSSCG00000027157	*SLC40A1*	Solute Carrier Family 40 Member 1	$-$0.365	1.23E$-$05	$-$0.462	3.19E$-$05	Downregulated
ENSSSCG00000006072	*VPS13B*	Vacuolar Protein Sorting 13 Homolog B	$-$0.359	4.30E$-$05	$-$0.469	5.52E$-$04	Downregulated
ENSSSCG00000028322	*BTG2*	BTG Anti-Proliferation Factor 2	$-$0.394	2.61E$-$09	$-$0.452	2.29E$-$04	Downregulated
ENSSSCG00000016059	*STAT4*	Signal Transducer And Activator Of Transcription 4	$-$0.354	6.36E$-$06	$-$0.43	6.75E$-$05	Downregulated
ENSSSCG00000028196	*MFF*	Mitochondrial Fission Factor	$-$0.352	6.40E$-$06	$-$0.427	3.85E$-$05	Downregulated

* In the cases where EdgeR detected a DE gene with high significance and the padj was not calculated in DESeq2 due to the presence of one outlier, we checked case by case and validated as DE, whenever suitable. The adjusted P-value showed in these cases is the value calculated prior to multitest for demonstration purposes only.

#### M. hyopneumoniae elicited an antioxidant response and induced the accumulation of NRF2 in the nuclei of NPTr cells

Among the up-regulated genes, we found several ones related to immune response and inflammation, such as C3 complement, *SAA3*, chemokines (*CXCL2* and *CCL20*) and galectins (*LGALS2* and *LGALS8*) (Supplementary Table S3). Interestingly, we also detected 64 up-regulated genes related to redox homeostasis and antioxidant defense (Table 3). We observed that 46 out of 64 have already been described as targets of the nuclear factor erythroid 2-related factor 2 (NRF2) in closely related species (Table 3). This transcription factor is involved in the protection of the cell against oxidative damage through transcription activation of cytoprotective genes^26, 27^. More specifically, several studies have shown the protective role of NRF2 in bacterial lung infections in rodents, being a critical factor for assembling the innate immune response in the host^28–31^.

**Table 3:** Up-regulated genes involved in redox homeostasis. Detailed information for differential expression of genes (from both the DeSeq2 and EdgeR methods) related to antioxidant and redox homeostasis functions. The great majority of such genes has been demonstrated to be activated by the NRF2 transcription factor in related species. Duplicated entries related to different transcripts of a given gene were deleted in this table, however calculation of the statistics provided in Supplementary Table S5 was made taking into account the total amount of 64 genes (including duplications).

DE genes			DESeq2		EdgeR		Reference$^{1}$
Gene ID	Associated gene name	Description	LogFC	padj	LogFC	FDR	
ENSSSCG00000028982	*AKR1C4*	Aldo-Keto Reductase Family 1 Member C4	0.869	2.61E$-$76	0.953	4.61E$-$54	Hayes and McMahon^117^
ENSSSCG00000025277	*AKR1CL1*	Aldo-Keto Reductase Family 1, Member C-Like 1	0.866	3.96E$-$76	0.949	5.46E$-$54	Hayes and McMahon^117^
ENSSSCG00000003402	*PGD*	Phosphogluconate Dehydrogenase	0.611	3.62E$-$61	0.647	2,71E$-$36	Hayes and Dinkova-Kostova^118^
ENSSSCG00000025273	*CYP11A1*$^{+}$	Cytochrome P450 Family 11 Subfamily A Member 1	0.510	1.39E$-$11	0.648	3.80E$-$11	
ENSSSCG00000010853	*EPHX1*	Epoxide Hydrolase 1	0.444	5.20E$-$15	0.503	1.76E$-$06	Hayes and Dinkova-Kostova^118^
ENSSSCG00000014540	*FTH1*	Ferritin Heavy Chain 1	0.436	1.81E$-$24	0.470	1.85E$-$14	Hayes and Dinkova-Kostova^118^
ENSSSCG00000003153	*FTL*	Ferritin Light Chain	0.427	1.25E$-$24	0.459	5.31E$-$14	Hayes and Dinkova-Kostova^118^
ENSSSCG00000000843	*TXNRD1*	Thioredoxin Reductase 1	0.426	7.45E$-$14	0.482	7.33E$-$13	Hayes and Dinkova-Kostova^118^
ENSSSCG00000002754	*NQO1*	NAD(P)H Quinone Dehydrogenase 1	0.415	7.89E$-$18	0.456	1.64E$-$12	Hayes and Dinkova-Kostova^118^
ENSSSCG00000002626	*GSTA1*$^{+}$	Glutathione S-Transferase A1	0.407	1.02E$-$12	0.461	4.18E$-$09	Hayes and Dinkova-Kostova^118^
ENSSSCG00000016312	*UGT1A6*$^{+}$	UDP Glucuronosyltransferase Family 1 Member A6	0.367	1.88E$-$18	0.394	7.49E$-$12	Hayes and Dinkova-Kostova^118^
ENSSSCG00000011147	*AKR1C1*	Aldo-Keto Reductase Family 1 Member C1	0.360	1.28E$-$04	0.511	1.20E$-$03	Hayes and Dinkova-Kostova^118^
ENSSSCG00000021386	*PTGR1*	Prostaglandin Reductase 1	0.358	6.02E$-$12	0.397	1.55E$-$04	Hayes and Dinkova-Kostova^118^
ENSSSCG00000001488	*GCLC*	Glutamate-Cysteine Ligase Catalytic Subunit	0.350	2.78E$-$10	0.392	2.89E$-$09	Hayes and Dinkova-Kostova^118^
ENSSSCG00000002825	*CES1*$^{+}$	Carboxylesterase 1	0.348	5.58E$-$12	0.384	2.93E$-$08	Hayes and Dinkova-Kostova^118^
ENSSSCG00000012136	*PIR*	Pirin	0.334	9.34E$-$07	0.392	7.21E$-$06	Brzóska et al.^119^
ENSSSCG00000025762	*GSR*$^{+}$	Glutathione S$-$Reductase	0.314	2.46E$-$03	0.489	2.57E$-$02	Hayes and Dinkova-Kostova^118^
ENSSSCG00000029144	*SRXN1*$^{+}$	Sulfiredoxin 1	0.309	3.31E$-$06	0.359	1.12E$-$05	Hayes and Dinkova-Kostova^118^
ENSSSCG00000010055	*GGT5*	Gamma-Glutamyltransferase 5	0.305	1.38E$-$03	0.421	3.33E$-$03	
ENSSSCG00000014833	*UCP2*	Uncopling Protein 2	0.303	5.10E$-$03	0.509	4.52E$-$02	
ENSSSCG00000028099	*SLC6A6*$^{+}$	Solute Carrier Family 6 Member 6	0.295	2.36E$-$03	0.412	5.50E$-$03	Hayes and McMahon^117^
ENSSSCG00000026759	*HMOX1*	Heme Oxygenase 1	0.272	9.35E$-$03	0.402	4.36E$-$02	Hayes and Dinkova-Kostova^118^
ENSSSCG00000008311	*CYP26B1*	Cytochrome P450 Family 26 Subfamily B Member 1	0.267	4.82E$-$03	0.359	8.22E$-$03	
ENSSSCG00000028996	*ALDH1A1*	Aldehyde Dehydrogenase 1 Family Member A1	0.261	1.47E$-$18	0.274	2.92E$-$07	Hayes and Dinkova-Kostova^18^
ENSSSCG00000006717	*PHGDH*	Phosphoglycerate Dehydrogenase	0.260	1.20E$-$07	0.285	4.62E$-$05	
ENSSSCG00000021067	*BLVRB*	Biliverdin Reductase B	0.259	2.00E$-$04	0.302	1.22E$-$03	Hayes and Dinkova-Kostova^118^
ENSSSCG00000013366	*LDHA*	Lactate Dehydrogenase A	0.255	9.07E$-$07	0.281	8.19E$-$05	
ENSSSCG00000029781	*SELENOM*	Selenoprotein M	0.254	2.25E0$-$2	0.406	5.78E$-$02	
ENSSSCG00000001723	*PLA2G7*	Phospholipase A2 Group VII	0.250	4.58E$-$04	0.294	2.13E$-$03	Hayes and Dinkova-Kostova^118^
ENSSSCG00000004093	*IYD*	Iodotyrosine Deiodinase	0.250	1.09E$-$02	0.339	1.83E$-$02	
ENSSSCG00000001963	*EGLN3*	Egl-9 Family Hypoxia Inducible Factor 3	0.248	7.36E$-$03	0.324	9.69E$-$03	
ENSSSCG00000003914	*PRDX1*	Peroxiredoxin 1	0.231	1.02E$-$07	0.249	1.27E$-$04	Hayes and Dinkova-Kostova^118^
ENSSSCG00000017092	*GPX3*	Glutathione Peroxidase 3	0.224	7.69E$-$05	0.250	1.89E$-$03	Kensler et al.^32^
ENSSSCG00000018048	*SLC5A10*	Solute Carrier Family 5 Member 10	0.215	3.21E$-$03	0.251	9.13E$-$03	Hayes and McMahon^117^
ENSSSCG00000003491	*AKR7A2*	Aldo-Keto Reductase Family 7 Member A2	0.205	1.69E$-$02	0.252	3.10E$-$02	Li et al.^120^
ENSSSCG00000021408	*TKT*	Transketolase	0.190	2.39E$-$06	0.204	1.49E$-$03	Hayes and Dinkova-Kostova^118^
ENSSSCG00000010340	*FAM213A*	Family With Sequence Similarity 213 Member A	0.184	1.62E$-$05	0.198	4.20E$-$03	
ENSSSCG00000010056	*GGT1*	Gamma-Glutamyltransferase 1	0.178	1.95E$-$02	0.209	2.50E$-$02	Hayes and Dinkova-Kostova^118^
ENSSSCG00000013030	*PRDX5*	Peroxiredoxin 5	0.177	1.04E$-$02	0.204	2.22E$-$02	Graham et al.^121^
ENSSSCG00000012847	*TALDO1*	Transaldolase 1	0.177	3.29E$-$03	0.199	2.33E$-$01	Hayes and Dinkova-Kostova^118^
ENSSSCG00000030461	*HEPHL1*	Hephaestatin Like 1	0.170	8.19E$-$05*	3.003	3.60E$-$04	
ENSSSCG00000018084	*ND3*	Mitochondrially Encoded NADH:Ubiquinone Oxidoreductase Core Subunit 3	0.166	2.19E$-$03	0.183	2.77E$-$02	
ENSSSCG00000012327	*HSD17B10*	Hydroxysteroid 17-Beta Dehydrogenase 10	0.164	1.66E$-$02	0.188	1.94E$-$02	
ENSSSCG00000003007	*BCKDHA*	Branched Chain Keto Acid Dehydrogenase E1, Alpha Polypeptide	0.163	3.52E$-$02	0.191	5.54E$-$02	
ENSSSCG00000010928	*KDM5B*	Lysine Demethylase 5B	0.161	2.36E$-$04	0.174	1.58E$-$02	
ENSSSCG00000014336	*EGR1*	Early Growth Response 1	0.159	2.52E$-$04	0.172	1.42E$-$02	Gomez et al.^31^
ENSSSCG00000008550	*SLC5A6*	Solute Carrier Family 5 Member 6	0.155	2.36E$-$03	0.170	2.44E$-$02	Hayes and McMahon^117^
ENSSSCG00000006324	*ALDH9A1*	Aldehyde Dehydrogenase 9 Family Member A1	0.155	6.71E$-$04	0.168	2.07E$-$02	
ENSSSCG00000022742	*PRDX6*	Peroxiredoxin 6	0.151	1.49E$-$03	0.164	2.28E$-$02	Hayes and Dinkova-Kostova^118^
ENSSSCG00000001701	*HSP90AB1*	Heat Shock Protein 90 Alpha Family Class B Member 1	0.149	2.11E$-$04	0.160	2.15E$-$02	Hayes and McMahon^117^
ENSSSCG00000028739	*CEBPB*	CCAAT/Enhancer Binding Protein Beta	0.148	1.32E$-$02	0.165	3.70E$-$01	Hayes and Dinkova-Kostova^118^
ENSSSCG00000004454	*ME1*	Malic Enzyme 1	0.145	5.30E$-$03	0.160	2.96E$-$01	Hayes and Dinkova-Kostova^118^
ENSSSCG00000010682	*PRDX3*	Peroxiredoxin 3	0.139	1.35E$-$02	0.154	7.06E$-$02	Gomez et al.^31^
ENSSSCG00000027525	*DHCR24*	24-Dehydrocholesterol Reductase	0.123	3.68E$-$03	0.133	5.52E$-$02	
ENSSSCG00000029876	*SOD2*	Superoxide Dismutase 2	0.112	1.66E$-$02	0.123	1.39E$-$01	Reszka et al.^122^

$^{1}$References related to genes regulated by NRF2.

$^{+}$Genes with more than one ID identified as DE.

* The P-value showed in these cases is the value calculated prior to multitesting for demonstration purposes only (see Table 2).

Indeed, *M. hyopneumoniae* infection induced the expression of genes related to the two biggest redox systems in NPTr cells - glutathione (*GGT1, GGT5, GGLC, GSR*) and thioredoxin (*TXNRD1, PRDX5, PRDX6*) -, and also genes coding for NADPH-regenerating enzymes (used by the aforementioned redox systems), as analogously reported for activation of NRF2 targets in mice^32^. Moreover, a number of antioxidant genes and genes coding for detoxification enzymes (such as the *AKR* gene family, *NQO1, HMOX* and *GST*) which were up-regulated during *M. hyopneumoniae* infection, were also reported to be activated by NRF2^32^ (Table 3). We performed a Fisher’s exact test to compare the proportion of genes within the genome expected to be targets of NRF2 with the proportion of up-regulated genes putatively regulated by this transcription factor. The results indicate an extremely significant correlation of an NRF2 target being an upregulated gene (p-value $< 0.00001$, Supplementary Table S5). The up-regulation of several targets of NRF2 was further validated by reverse-transcription quantitative PCR (RT-qPCR) and the results are available in Supplementary Fig. S3.

One of the many compounds known to activate the NRF2 pathway is hydrogen peroxide^33^. The production of hydrogen peroxide was previously described to have an important cytotoxic effect in several *Mycoplasma* species, such as *M. pneumoniae*^34^ and *M. mycoides* subsp. *mycoides*^35^. More specifically, the cytotoxity of *M. mycoides* subsp. *mycoides* was correlated to the bacterium’s ability to translocate hydrogen peroxide directly into the host cell^36^. Previously, we have identified that *M. hyopneumoniae* was capable of producing this toxic product from glycerol metabolism^5^. Although the production of hydrogen peroxide by the *M. hyopneumoniae* strain J was not detected in that work, novel analyses indicate that in conditions where glucose is scarce, the attenuated strain J is able to produce this toxic metabolite (Supplementary Fig. S4). In addition, we were able to detect hydrogen peroxide in the medium of the NPTr cells infected with *M. hyopneumoniae* in the presence of glycerol (Supplementary Fig. S4C). Although the production was higher in the cells infected with the pathogenic strain 7448, *M. hyopneumoniae* strain J was also able to produce the toxic product, indicating that both strains could potentially cause oxidative damage to the host cells. These results are in accordance with the gene expression of the putative enzyme responsible for hydrogen peroxide production (GlpO) in *M. hyopneumoniae* strain J, which did not differ from the pathogenic strains^5^. During infection in general, bacteria need to compete with host cells (and other organisms in the environment) for glucose and other energy sources. For instance, in *M. pneumoniae*, Halbedel et al. (2004)^37^ showed that even though glucose was the most efficient carbon source for biomass yield (as is the case for *M. hyopneumoniae*), the authors propose that glycerol is not only a carbon source, but it could be used by this species as an indicator that they reached their preferred ecological niche, a lipid-rich mucosal surface. Thus, it is plausible to say that *M. hyopneumoniae* also uses glycerol as a carbon source *in vivo*, however whether this is a result of competition against other fast-growing bacteria or if this species is targeting a glycerol-rich niche, we cannot affirm at this point. What we can affirm is that glycerol is not the most efficient carbon source for both *M. pneumoniae* and *M. hyopneumoniae*, and yet these species make use of it for energy uptake and also for other advantages, such as hydrogen peroxide production. Thus, it is possible that the hydrogen peroxide produced by *M. hyopneumoniae* strain J as a result of glycerol metabolism might be one of the triggers that activated the transcription factor NRF2.

**Figure 2:**
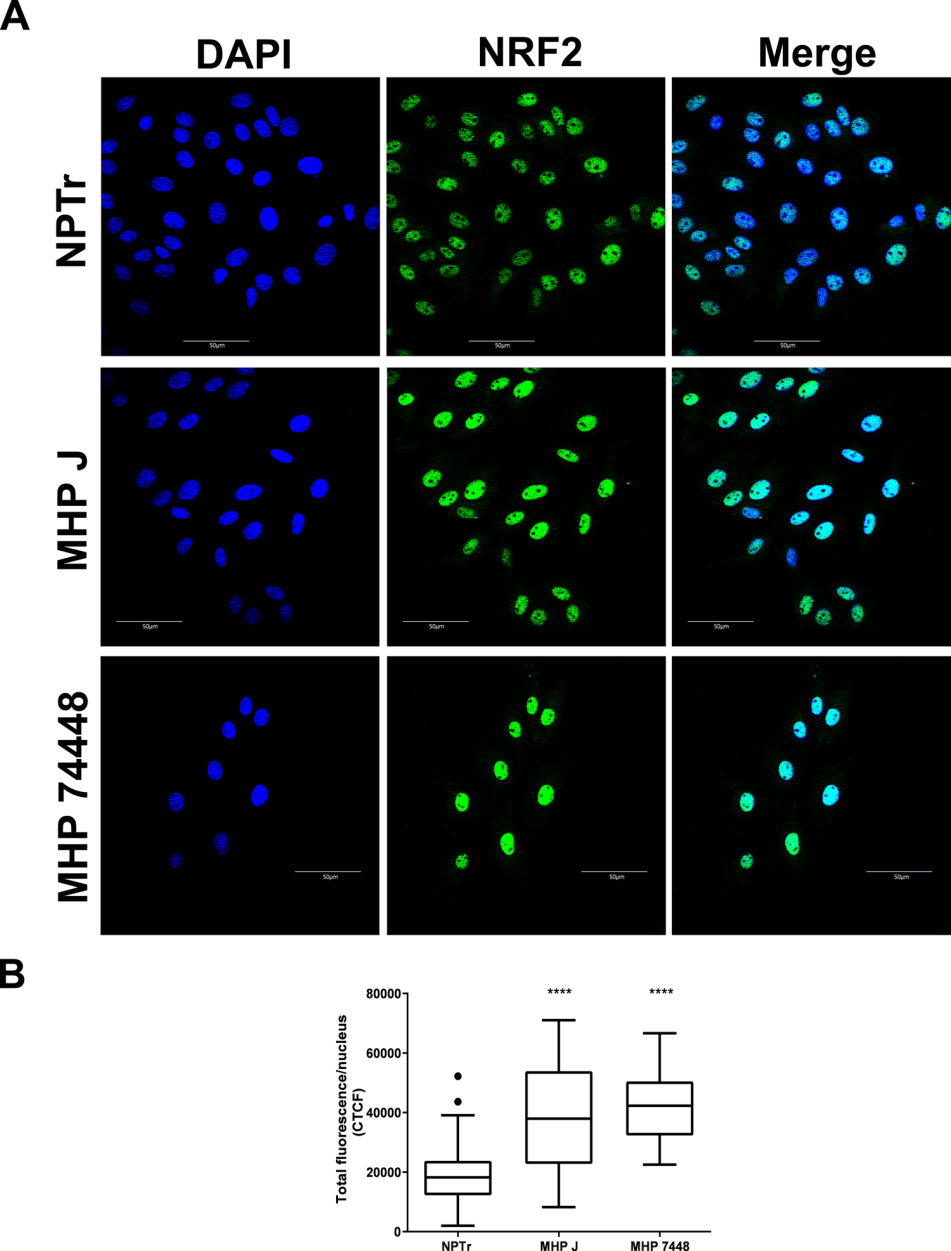
Localization and expression pattern of the NRF2 protein in cells infected with *M. hyopneumoniae.***A.** Results of the immunofluorescence microscopy analysis indicating the accumulation of NRF2 in the nuclei of infected cells after 1 h of incubation. Both attenuated (J) and virulent (7448) strains of *M. hyopneumoniae* induced the accumulation of the transcription factor in the nuclei of the epithelial cells. Eukaryotic cell NRF2 was labeled with anti-NRF2 antibody (green) and nuclei were stained with DAPI (blue). Scale bars: $50\,\mu \hbox {m}$. **B.** Total NRF2 green fluorescence per nucleus of control and infected cells. Both *M. hyopneumoniae* strains significantly increased the concentration of NRF2 in the nuclei of infected cells. Boxplots represent the measures of at least 25 nuclei in each condition. Outliers are represented as black dots. CTCF—corrected total cell fluorescence. NPTr - uninfected cells. MHP J - NPTr cells infected with *M. hyopneumoniae* strain J. MHP 7448 - NPTr cells infected with *M. hyopneumoniae* strain 7448. **** $\hbox {p} < 0.0001.$

It has been demonstrated in other species that NRF2 is largely regulated at the post-transcriptional level by its sub-cellular distribution, which is controlled by the Kelch-like ECH-associated protein (KEAP1). Under normal conditions, a portion of NRF2 is retained in the cytoplasm via its interaction to KEAP1 and it is subsequently ubiquitinated and degraded by the proteasome. In response to oxidative stress, reactive cysteines in KEAP1 are modified, generating conformational changes in the complex and releasing NRF2, which is translocated and accumulates into the nucleus^27, 32, 38^. In our analysis, we did not detect a difference in the expression of the mRNAs of NRF2 (base mean expression around 8000 reads, a logFC between infected and non-infected conditions close to zero and a non-significant adjusted p-value of 0.99) and KEAP1 (base mean expression around 4500 reads, logFC between infected and non-infected conditions close to zero and non-significant adjusted p-value of 0.94). However, we were able to demonstrate by confocal immunofluorescence microscopy (Fig. 2) that both the attenuated and the virulent strains of *M. hyopneumoniae* induced a statistically significant accumulation of this transcription factor in the nuclei of NPTr cells (Fig. 2B). In the nucleus, NRF2 is able to recognize and bind to antioxidant response element (ARE) motifs in the promoter region of target genes, activating their transcription^39^. In this work, we detected at least one conserved ARE sequence upstream the start codon in the promoter regions of 44 out of the 46 NRF2 predicted targets (with stringent search to TG/TAnnnnGC) with the use of fuzznuc software from EMBOSSv6.6.0 package^40^ (Supplementary Table S6).

In this way, it seems that *M. hyopneumoniae* infection has the potential to cause oxidative stress to the host cells, which in turn activate antioxidant response genes induced by NRF2 to fight the infection and maintain cellular homeostasis. We believe that this oxidative stress was in part related to the hydrogen peroxide produced by this bacterium, although more experiments are needed to prove the association between this mechanism and the up-regulation of antioxidant response genes.

#### M. hyopneumoniae induced down-regulation of cytoskeleton and ciliary genes as well as a decrease of actin stress fibers in NPTr cells

We identified several down-regulated genes related to ciliary function, cytoskeleton and cell cycle/cell division. The impairment of the ciliary motility is a well-known effect caused by several *Mycoplasma* respiratory species^41^, such as *M. pneumoniae* and *M. gallisepticum*^42, 43^. It is also well established that *M. hyopneumoniae* attaches to cilia of epithelial cells and promotes ciliostasis and loss of cilia, causing damage to the mucociliary apparatus^4, 24^. Therefore, our hypothesis is that one of the reasons for epithelial damage, besides physical adhesion, could be associated with modulations in gene expression induced by the infection, which is a running hypothesis for at least two epithelial pathogens from the genus *Mycobacterium*^44^. In agreement with this hypothesis, we were able to identify the down-regulation of genes coding for axonemal dyneins (*DNAH11, DNAH12, DNAI2, DNAL1*), which are essential for the ciliary motility^45^. In addition, genes necessary for axonemal dynein assembly (*DYX1C1*)^46^, genes related to ciliogenesis (*CEP162, DCDC2, MACF1, IFT57*)^47–50^, ciliary polarization (*INTU*)^51^, ciliary beating (*MYO1D*)^52^ and several others in which the mutation or knockout is associated with ciliopathies (*LRRC6, MNS1, AK7*)^53–55^ were down-regulated in the infected cells (Table 4). Interestingly, in line with our results, a recent study compared the transcriptional response of unvaccinated and vaccinated chicken infected with *M. gallisepticum* and the authors identified enrichment of GO terms in down-regulated genes related to cilia and cytoskeleton in unvaccinated animals^56^. Protein functions encoded by the top down-regulated genes were involved in microtubule assembly and stability, axonemal dynein complex assembly, and formation and motor movement of cilia, indicating that at least in one *Mycoplasma* species the ciliary damage caused by infection could be also explained by the down-regulation of genes involved in the ciliary function.

**Table 4:** Down-regulated genes involved in cytoskeleton and cilia.

DE genes			DESeq2		EdgeR	
Gene ID	Associated gene name	Description	LogFC	P-adj	LogFC	P-adj
ENSSSCG00000011441	*TNNC1*	Troponin C1, Slow Skeletal And Cardiac Type	$-$0.399	5.92E$-$05	$-$0.633	3.27E$-$03
ENSSSCG00000001791	*SAXO2*	Stabilizer Of Axonemal Microtubules 2	$-$0.328	2.08E$-$03	$-$0.547	4.38E$-$02
ENSSSCG00000014191	*FER*	FER Tyrosine Kinase	$-$0.331	1.75E$-$04	$-$0.426	4.07E$-$04
ENSSSCG00000015036	*DIXDC1*	DIX Domain Containing 1	$-$0.352	3.48E$-$07	$-$0.405	2.57E$-$06
ENSSSCG00000009079	*INTU*	Inturned Planar Cell Polarity Protein	$-$0.273	9.94E$-$03	$-$0.403	4.79E$-$02
ENSSSCG00000015531	*CEP350*	Centrosomal Protein 350	$-$0.314	3.28E$-$04	$-$0.400	2.58E$-$03
ENSSSCG00000024357	*DNAI2*	Dynein Axonemal Intermediate Chain 2	$-$0.276	7.42E$-$03	$-$0.395	4.04E$-$02
ENSSSCG00000002347	*DNAL1*	Dynein Axonemal Light Chain 1	$-$0.285	2.87E$-$03	$-$0.378	8.99E$-$03
ENSSSCG00000004705	*MAP1A*	Microtubule Associated Protein 1A	$-$0.271	7.62E$-$03	$-$0.376	2.25E$-$02
ENSSSCG00000001728	*CD2AP*	CD2 Associated Protein	$-$0.330	5.69E$-$08	$-$0.366	8.97E$-$05
ENSSSCG00000000874	*GAS2L3*	Growth Arrest Specific 2 Like 3	$-$0.270	5.65E$-$03	$-$0.361	1.70E$-$02
ENSSSCG00000010896	*ASPM*	Abnormal Spindle Microtubule Assembly	$-$0.291	3.90E$-$05	$-$0.332	3.79E$-$02
ENSSSCG00000015329	*PPP1R9A*	Protein Phosphatase 1 Regulatory Subunit 9A	$-$0.245	1.54E$-$02	$-$0.328	3.17E$-$02
ENSSSCG00000016542	*LRGUK*	Leucine Rich Repeats And Guanylate Kinase Domain Containing	$-$0.230	2.63E$-$02	$-$0.309	5.49E$-$02
ENSSSCG00000016725	*TNS3*	Tensin 3	$-$0.239	1.28E$-$02	$-$0.306	2.94E$-$02
ENSSSCG00000016608	*IQUB*	IQ Motif And Ubiquitin Domain Containing	$-$0.248	2.68E$-$03	$-$0.295	1.01E$-$02
ENSSSCG00000002780	*TPPP3*	Tubulin Polymerization Promoting Protein Family Member 3	$-$0.251	1.52E$-$03	$-$0.294	8.99E$-$03
ENSSSCG00000006394	*CFAP45*	Cilia And Flagella Associated Protein 45	$-$0.251	1.56E$-$03	$-$0.294	6.49E$-$03
ENSSSCG00000013332	*KIF18A*	Kinesin Family Member 18A	$-$0.227	2.00E$-$02	$-$0.290	3.88E$-$02
ENSSSCG00000021571	*KIF27*	Kinesin Family Member 27	$-$0.246	1.78E$-$03	$-$0.287	9.15E$-$03
ENSSSCG00000020990	*DNAH12*	Dynein Axonemal Heavy Chain 12	$-$0.235	6.82E$-$03	$-$0.283	1.75E$-$02
ENSSSCG00000000530	*FGD4*	FYVE, RhoGEF and PH domain containing 4	$-$0.231	7.32E$-$03	$-$0.277	3.30E$-$02
ENSSSCG00000003931	*KIF2C*	Kinesin Family Member 2C	$-$0.253	4.30E$-$05	$-$0.276	3.27E$-$02
ENSSSCG00000015523	*RALGPS2*	Ral GEF With PH Domain And SH3 Binding Motif 2	$-$0.255	1.06E$-$06	$-$0.270	3.30E$-$02
ENSSSCG00000008768	*ARAP2*	ArfGAP With RhoGAP Domain, Ankyrin Repeat And PH Domain2	$-$0.220	7.54E$-$03	$-$0.256	3.11E$-$02
ENSSSCG00000006936	*ODF2L*	Outer Dense Fiber Of Sperm Tails 2 Like	$-$0.213	1.57E$-$02	$-$0.255	4.25E$-$02
ENSSSCG00000001869	*PEAK1*	Pseudopodium Enriched Atypical Kinase 1	$-$0.210	1.58E$-$02	$-$0.250	3.89E$-$02
ENSSSCG00000011941	*IFT57*	Intraflagellar Transport 57	$-$0.223	3.97E$-$04	$-$0.242	2.12E$-$03
ENSSSCG00000011745	*PRKCI*	Protein Kinase C Iota	$-$0.225	5.70E$-$05	$-$0.239	8.27E$-$02
ENSSSCG00000002504	*AK7*	Adenylate Kinase 7	$-$0.209	2.77E$-$03	$-$0.231	4.20E$-$03
ENSSSCG00000017884	*TEKT1*	Tektin 1	$-$0.199	1.40E$-$02	$-$0.229	5.40E$-$02
ENSSSCG00000005952	*LRRC6*	Leucine Rich Repeat Containing 6	$-$0.199	1.28E$-$02	$-$0.227	3.64E$-$02
ENSSSCG00000005078	*DAAM1*	Dishevelled Associated Activator Of Morphogenesis 1	$-$0.206	1.97E$-$03	$-$0.225	8.79E$-$03
ENSSSCG00000009629	*BIN3*	Bridging Integrator 3	$-$0.195	1.39E$-$02	$-$0.221	3.88E$-$02
ENSSSCG00000015379	*DNAH11*	Dynein Axonemal Heavy Chain 11	$-$0.204	1.68E$-$03	$-$0.221	3.21E$-$03
ENSSSCG00000016658	*ANLN*	Anillin Actin Binding Protein	$-$0.212	1.05E$-$05	$-$0.220	1.89E$-$03
ENSSSCG00000016794	*MYO10*	Myosin X	$-$0.210	6.68E$-$05	$-$0.220	3.93E$-$03
ENSSSCG00000004969	*KIF23*	Kinesin Family Member 23	$-$0.205	6.08E$-$05	$-$0.214	4.68E$-$02
ENSSSCG00000003654	*MACF1*	Microtubule-Actin Crosslinking Factor 1	$-$0.201	4.70E$-$04	$-$0.214	1.08E$-$02
ENSSSCG00000015570	*IVNS1ABP*	Influenza Virus NS1A Binding Protein	$-$0.205	1.05E$-$05	$-$0.212	1.91E$-$02
ENSSSCG00000004289	*CEP162*	Centrosomal Protein 162	$-$0.191	1.01E$-$02	$-$0.212	3.17E$-$02
ENSSSCG00000010457	*KIF20B*	Kinesin Family Member 20B	$-$0.192	1.92E$-$03	$-$0.206	4.54E$-$02
ENSSSCG00000010351	*CCSER2*	Coiled-Coil Serine Rich Protein 2	$-$0.201	2.27E$-$06	$-$0.206	2.70E$-$03
ENSSSCG00000007235	*TPX2*	TPX2, Microtubule Nucleation Factor	$-$0.189	3.76E$-$04	$-$0.198	4.85E$-$02
ENSSSCG00000017728	*MYO1D*	Myosin ID	$-$0.184	5.05E$-$05	$-$0.189	2.22E$-$02
ENSSSCG00000010471	*KIF11*	Kinesin Family Member 11	$-$0.179	5.69E$-$04	$-$0.186	2.09E$-$02
ENSSSCG00000016781	*TRIO*	Trio Rho Guanine Nucleotide Exchange Factor	$-$0.174	1.51E$-$03	$-$0.182	2.87E$-$02
ENSSSCG00000009348	*STARD13*	StAR Related Lipid Transfer Domain Containing 13	$-$0.174	4.69E$-$04	$-$0.180	2.09E$-$02
ENSSSCG00000009793	*CLIP1*	CAP-Gly Domain Containing Linker Protein 1	$-$0.174	1.19E$-$04	$-$0.178	9.64E$-$03
ENSSSCG00000001085	*DCDC2*	Doublecortin Domain Containing 2	$-$0.162	2.19E$-$03	$-$0.168	3.95E$-$02
ENSSSCG00000005235	*KANK1*	KN Motif And Ankyrin Repeat Domains 1	$-$0.163	2.54E$-$04	$-$0.166	5.13E$-$02
ENSSSCG00000009125	*ANK2*	Ankyrin 2	$-$0.159	4.57E$-$03	$-$0.166	4.54E$-$02
ENSSSCG00000010313	*VCL*	Vinculin	$-$0.159	1.43E$-$05	$-$0.159	1.20E$-$02
ENSSSCG00000016061	*MYO1B*	Myosin IB	$-$0.150	1.13E$-$03	$-$0.152	5.52E$-$02

Detailed information for differential expression of genes (from both the DeSeq2 and EdgeR methods) related to cytoskeleton and ciliary functions.

**Figure 3:**
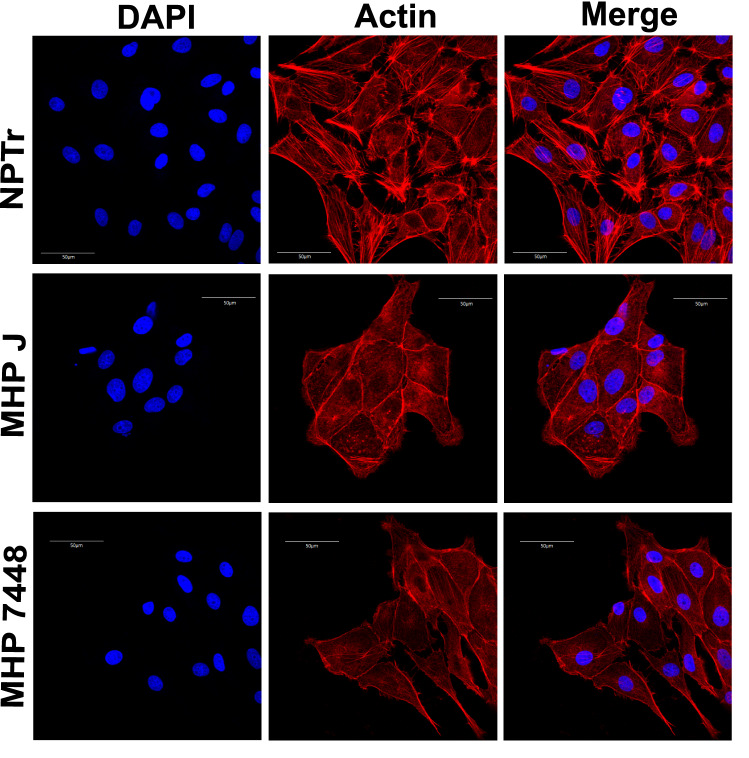
Organization of actin fibers in cells infected with *M. hyopneumoniae.* Results of the immunofluorescence microscopy analysis indicating the reduction and change in the pattern of actin stress fibers in infected cells. Eukaryotic cell actin was labeled with phalloidin (red) and nuclei were stained with DAPI (blue). Both attenuated (J) and virulent (7448) strains of *M. hyopneumoniae* altered the organization and abundance of actin fibers after 24 h of infection, however this effect can already be seen after 1 h of incubation (not shown). NPTr - uninfected cells. MHP J - NPTr cells infected with *M. hyopneumoniae* strain J. MHP 7448 - NPTr cells infected with *M. hyopneumoniae* strain 7448. Scale bars: $50\,\mu \hbox {m}.$

Besides ciliary genes, we also detected the down-regulation of cytoskeleton-related genes, both from microtubules and actin filaments, involved in the organization, rearrangement and stability of these structures (Table 4). It was previously described that the intracellular species *M. penetrans* is able to trigger reorganization of the host cell cystoskeleton, promoting aggregation of tubulin and $\alpha$-actinin and condensation of phosphorylated proteins^57^. To investigate if *M. hyopneumoniae* indeed affected the host cell cytoskeleton, we verified the organization of actin fibers in infected cells by confocal immunofluorescence microscopy. The actin stress fibers were notably less evident in the infected cells, as opposed to the control condition, in which they were abundant and more evenly distributed (Fig. 3). However, whenever present in the infected conditions, these stress fibers were either disorganized and/or at the periphery of the cells. These results corroborate studies from Raymond et al. (2018)^58^, which suggested that this species induces cytoskeletal rearrangements in the porcine respiratory tract. Although actin is not a major component of the ciliary axoneme, actin cytoskeleton has been implicated in every stage of ciliogenesis and many aspects of ciliary function^59^, directly associating these two down-regulated functions. In addition, it has recently been shown that *M. hyopneumoniae* expresses surface-accessible actin-binding proteins and that the host’s extracellular actin may act as a receptor for this bacterium in PK-15 epithelial cells^60^, indicating its importance for successful infection. Furthermore, the reduction of visible actin stress fibers caused by *M. hyopneumoniae* may also be related to the activation of NRF2, since the actin cytoskeleton is a scaffold necessary to maintain the transcription factor in the cytoplasm^61^.

We also identified the down-regulation of cytoskeleton-related genes that play a role during cell division, such as *BUB1B, CENP-I, NEK2* and *SPAG5* (Supplementary Table S3). Many of these genes are involved with the mitotic spindle and chromosome segregation^62–69^. Genes encoding microtubule dependent motor proteins that physically affect chromosome segregation, such as kinesins (usually up-regulated during mitosis)^70^, as well as genes related to cell cycle progression were down-regulated during infection (Supplementary Table S3). These results suggest a repression of cell division of infected cells. The manipulation of cell cycle by pathogens has been extensively reported, with different pathogens being able to arrest different points of the cell cycle^71–74^. Therefore, it is possible that *M. hyopneumoniae* infection also interferes with the host cell cycle.

### miRNA expression profiles

A total of 14 small RNA (sRNA) libraries were generated with the Illumina HiSeq2500 platform (Supplementary Table S7). A complete description of the samples is provided in Table 1 and Fig. S2. The raw reads were submitted to the NCBI Sequence Read Archive under accession number PRJNA545822. After removing adapters and filtering low quality reads, sRNA-seq yielded from 1 to 21 million single-end clean reads. Trimming and mapping information for each sRNA sample is given in Supplementary Table S7.

For intracellular sRNAs (INTRA: samples S1 to S6), we were able to map around 93% of the reads against the porcine genome. We also mapped all sRNA reads against the *M. hyopneumoniae* strain 7448 genome and samples had no more than 0.05% of unique mycoplasmal reads (Supplementary Table S7). All intracellular sRNA samples had similar sequencing depth and showed a typical miRNA distribution length curve ranging from 18 to 26 nt, with a peak at 22 nt (Supplementary Fig. S5A). We also performed a homology analysis of raw reads and we provide the percentage of intracellular clean reads clustered by classical types of sRNAs (Supplementary Fig. S5B), showing an enrichment of miRNA-homolog reads (around 40%).

As expected, total extracellular sRNA samples (EXTRA: S7 to S10) had more RNA degradation due to the presence of RNAses and degraded mRNAs in the extracellular environment. Extracellular sRNA samples of cells infected with the bacterium (S9 and S10) had up to 20% of reads mapped to *M. hyopneumoniae*, as they were presumed to have more remnants of mycoplasmal cells (Supplementary Table S7). However, the great majority of reads mapping to the bacteria were mainly product of mRNA degradation, as they did not have any specific signature for sRNAs when compared to previous results from Siqueira et al. (2016)^75^. In this way, we filtered out (i) sequences $< 18$ nt and (ii) sequences mapped to the *M. hyopneumoniae* genome. Sample S8 (EXTRA) had a sequencing depth smaller than its replicate (S7); however, the distribution of counts was overall similar between replicates and we included it in the further analyses.

Extracellular exosomal sRNA samples (EXO: S11 and S12) had some RNA degradation ($<18$ nt), but maintained a pronounced peak at 22 nt. Extracellular sRNAs from vesicle-free supernatant (SN: samples S13 and S14) were more problematic, probably due to (i) too many pre-processing steps and (ii) the presence of RNAses in the extracellular environment. Sample S14 did not yield a minimum amount of reads necessary for the subsequent steps and this condition (SN) was not used for further analyses.

#### Annotation of known and novel miRNAs

In order to perform miRNA prediction with mirDeep2^76^, we only took into account reads from intracellular samples. We predicted a total of 1,041 miRNAs, which were further clustered into 773 groups. From these, 478 were completely novel (Supplementary Table S8). We created a *Sus scrofa* miRNA database (ssc-miRNA-DB) with 1,906 different entries from three different sources: 411 known miRNAs in *Sus scrofa* from miRBase (release 21)^77^, 722 annotated by Martini et al., (2014)^78^ and the 773 clusters of mature miRNAs predicted by our analysis. More than 50% of intracellular sRNA clean reads were aligned against the ssc-miRNA-DB (Supplementary Table S7). All other samples were also mapped against this database. The complete pipeline used in this study for miRNA prediction is described in Supplementary Fig. S6.

#### miRNA differential expression

In all sRNA samples, we detected (with at least 10 counts across all libraries) 290 from the 411 miRNAs from miRBase and 214 of the miRNAs described by Martini et al., (2014)^78^. Also, 263 predicted miRNAs had more than 10 counts across all libraries. We only took into account the 491 miRNAs that had more than 50 counts across all libraries for differential expression analysis (Supplementary Table S9).

In total, we identified 170 DE miRNAs (121 up-regulated, 48 down-regulated and 1 ambiguous). Table  5 shows 10 selected up-regulated and down-regulated miRNAs detected in this study, and the complete list of DE miRNAs is summarized in Supplementary Table S10. Several homologs of these miRNAs have already been linked to bacterial infection response or immune system response in the literature and these references are listed in Table 5. With the exception of one ambiguous case (ssc-miR-9842-5p), whenever a miRNA was detected as DE in more than one condition (INTRA, EXTRA or EXO), the change in expression (either up- or down-regulated) was in accordance between them (Supplementary Table S10).

**Table 5:** Selected DE miRNAs. Information about selected up- and down-regulated miRNAs in intracellular, extracellular and exosome samples.

miRNA ID	DB Type	Intracellular		Extracellular		Exosomes		Play a role in other bacterial infection
		DE	LogFC	DE	LogFC	DE	GFOLD	
Novel-Chr13-miR-10	novel	Upregulated	4.121$^{*1}$	Upregulated	9.420	Upregulated	3.659	
Novel-Chr4-miR-57	novel	Upregulated	1.218	Upregulated	12.164	Upregulated	11.582	
Novel-Chr9-miR-16	novel	Upregulated	0.768	Upregulated	13.079	Upregulated	7.947	
Novel-Chr9-dna-26	novel	Upregulated	1.448	Upregulated	11.270	Upregulated	3.592	*Pseudomonas aeruginosa*^123^$$^{*2}$$
mmu-mir-2143-2_6_251367_251451_-_3p_-5-424-HC	Martini et al., 2014	Upregulated	2.053$^{*1}$	Upregulated	5.899	Upregulated	2.187	
ssc-miR-1285	mirBase	NS		Upregulated	4.965	NS		*Chlamydia trachomatis*^124, 125^
ssc-miR-196b-5p	mirBase	NS		Upregulated	3.971	NS		*Mycobacterium avium*^126^
ssc-miR-212	mirBase	NS		Upregulated	8.723	NS		*Mycobacterium tuberculosis*^127^
ssc-miR-24-1-5p	mirBase	NS		Upregulated	9.742	NS		*Escherichia coli* and *Staphylococcus aureus*^128^
ssc-miR-146a-5p	mirBase	Upregulated	1.015	ND		ND		*Helicobacter pylori*^129–131^ LPS stimulation^132^
ssc-miR-9842-5p	mirBase	NS		Upregulated	4.484	Downregulated	$-$2.497	
ssc-miR-184	mirBase	Downregulated	$-$0.594	ND		NS		*Chlamydia trachomatis*^133^
ssc-miR-140-3p	mirBase	NS		NS		Downregulated	$-$3.536	*Mycobacterium tuberculosis*^134^
ssc-miR-769-3p	mirBase	NS		Downregulated	$-$3.924$^{*1}$	NS		
ssc-miR-101	mirBase	NS		Downregulated	$-$4.154$^{*1}$	Downregulated	$-$2.986	*Helicobacter pylori*^135^
ssc-miR-107	mirBase	NS		Downregulated	$-$2.515$^{*1}$	Downregulated	$-$2.052	Gut microbiota^136^
ssc-miR-31	mirBase	NS		Downregulated	$-$1.167$^{*1}$	Downregulated	$-$2.004	*Helicobacter pylori*^135^ T-cell activation^137^
ssc-miR-532-5p	mirBase	NS		Downregulated	$-$3.343	Downregulated	$-$3.916	LPS stimulation^132^
antisense-pn8/bta-mir-2320_6_43886629_43886722_-_NA-424-HC	Martini et al., 2014	NS		Downregulated	$$-$$3.150$$^{*1}$$	Downregulated	$$-$$4.440	*Actinobacillus pleuropneumoniae*^138^$$^{*3}$$
antisense-ssc-mir-320a*_14_6473893_6473973_-_NA;antisense- sscmir-320a*_14_6473894_6473960_-_NA-424-HC	Martini et al., 2014	NS		Downregulated	$-$1.907$^{*1}$	Downregulated	$-$2.933	*Helicobacter pylori*^139^
ssc-mir-107-shorter/ssc-isomir-107_14_106321702_106321788_-_ NA-424-HC	Martini et al., 2014	NS		Downregulated	$-$2.582$^{*1}$	Downregulated	$-$2.206	Gut microbiota^136^

Several homologues of these miRNAs were already described to be involved with bacterial infection in other species, and references are provided whenever we found a correlation in the same direction of expression as in this work. The only miRNA that showed ambiguous expression among conditions was ssc-miR-9842-5p, which had inverse expression between the extracellular (up-regulated) and exosome (down-regulated) samples. NS: Not significant; ND: Not detected.

$^{*1}$: Whenever LogFC from DESeq2 was not available, we provide LogFC from EdgeR;

$^{*2}$: Reference related to cel-miR-233-5p, a possible homolog of novel miRNA 9-dna-26;

$^{*3}$: *A. pleuropneumoniae* was also related to a swine cell infection.

#### Targets of DE miRNAs were enriched in genes related to redox homeostasis, translation and cytoskeleton

In order to better understand the biological functions that could be involved with the 170 DE miRNAs, we performed different analyses to predict their potential targets. In this sense, all DE miRNAs had at least one DE gene as a predicted target. The complete pipeline used for target prediction in this study is provided in Supplementary Fig. S7.

**Figure 4:**
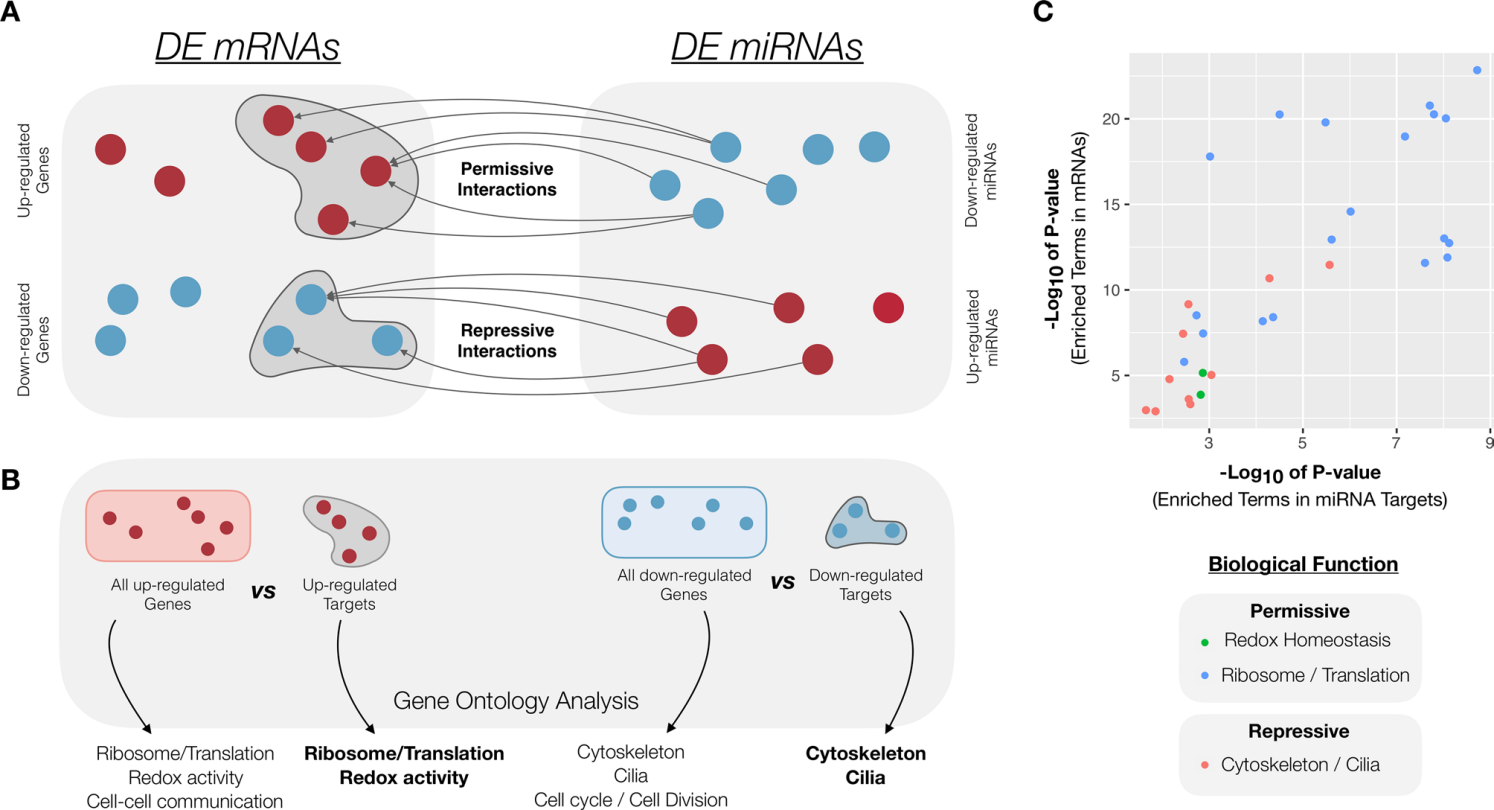
Correlation of GO terms between DE mRNAs and targets of DE miRNAs. **A** A permissive interaction occurs between a down-regulated miRNA (depicted in blue) and an up-regulated mRNA (depicted in red), whereas a repressive interaction is one in which the miRNA is up-regulated (red) and its target mRNA is down-regulated (blue). **B.** We performed GO enrichment analyses with the complete up- and down-regulated lists of mRNAs and also with the subset of miRNA targets among each of these lists. **C.** A correlation of some of the GO terms from DE mRNAs and targets of DE miRNAs was detected, indicating that these functions might also be regulated by miRNAs.

We predicted a total of 79,276 interaction pairs between miRNAs and mRNAs. In this way, based only on the predictions of the software used here, a miRNA could potentially target, on average, 465 genes in the entire porcine genome. However, we only considered as targets the mRNAs that were detected as DE in this study, which significantly decreased our list to a total of 4,287 interaction pairs. We chose to focus on anticorrelations between miRNA and mRNA expression (and kept 1,939 interaction pairs), as the main mode of action of miRNAs is a destabilization of mRNAs^79^ and the fact that most of experimental validations in the literature are related to interaction pairs with inversed regulation. In this context, a permissive interaction is generally described as one that occurs between a down-regulated miRNA and an up-regulated mRNA, while a repressive interaction is one in which the miRNA is up-regulated with consequent down-regulation of the target mRNA^78^ (Fig. 4A). In our results, permissive interactions represented 267 genes and 50 miRNAs, while repressive interactions occurred between 425 genes and 121 miRNAs, accounting for 598 permissive and 1341 repressive interactions between DE mRNAs and miRNAs. Supplementary Table S11 summarizes the permissive and repressive target pairs predicted in this study.

We performed GO analyses to compare the DE genes and targets of DE miRNAs in order to investigate whether their functions could be correlated (Fig. 4B and Supplementary Fig. S8). Interestingly, we were able to identify a correlation between the enriched terms in miRNA targets with some of the GO terms detected in the mRNA up-regulated or down-regulated GO results (Fig. 4C). Target genes from permissive interactions were associated with ribosome/translation and oxidation-reduction activity, whereas target genes from the repressive interactions had enriched terms related to cytoskeleton and ciliary function (Fig. 4C). It is important to highlight the relevance of the miRNAs found in exosome-like vesicles and in extracellular samples in the identification of GO enriched terms of the miRNA targets, since only a small part of the permissive and repressive interactions involved intracellular miRNAs. Complete GO enrichemnt of the miRNA targets is found in Supplementary Table S12.

#### Regulatory network reconstruction and analysis

In order to gain a broader view of the host’s response to the presence of *M. hyopneumoniae*, we built a general regulatory network by integrating mRNA gene expression with predicted miRNA-mRNA interactions collected and analyzed in this work (Supplementary Fig. S9). We also included information about physical and genetic validated interactions from the BioGRID v3.4 database^80, 81^. As previously mentioned, at this point we only took into account interaction pairs that were either repressive or permissive. The global interaction network was composed by 774 nodes and 1965 arcs and our objective was to identify miRNA-mRNA expression patterns. We performed a functional analysis with the use of CLueGO^82^ and we also detected within the permissive interactions the enrichment of several processes related to immune response and inflammation (Supplementary Fig. S10).

**Figure 5:**
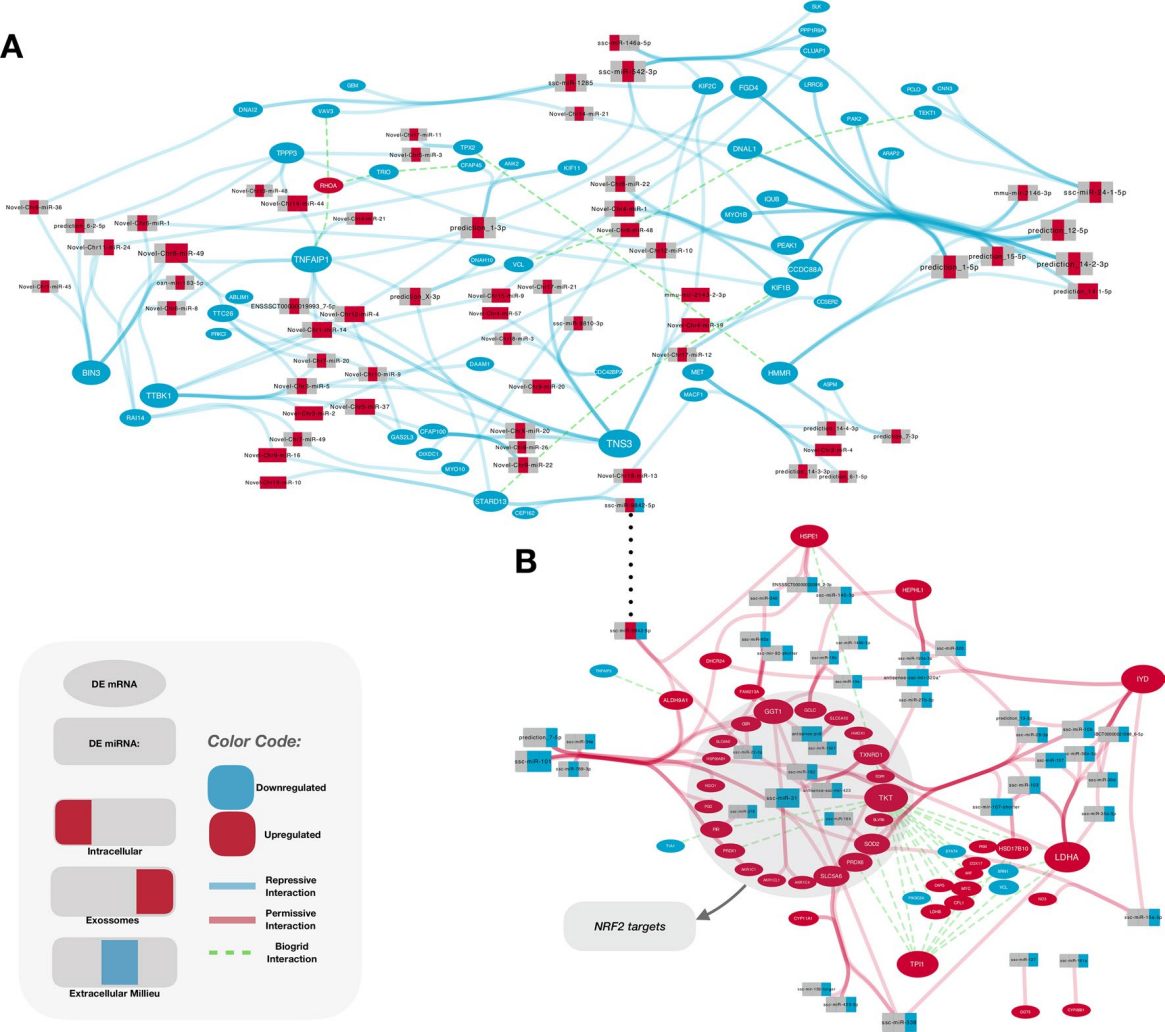
Interaction networks of DE miRNAs and genes involved with cytoskeleton/cilia and redox homeostasis. Rectangles depict miRNAs and ellipses represent genes. Blue ellipses/rectangles indicate that the gene/miRNA was down-regulated and red indicate gene/miRNAs detected as up-regulated. BioGrid interactions are seen as green dashed arcs. These represent both physical and genetic interactions between either genes or proteins from such genes. **A.** Cytoskeleton/cilia interaction network. We detected many repressive interactions involving genes related to cytoskeleton and ciliary function (blue arcs). **B.** Redox homeostasis interaction network. Permissive interactions of genes related to redox homeostasis are shown as red arcs. The great majority of the miRNAs targeting these genes are DE only in exosomes (rectangles partially colored in blue, far right). NRF2 targets: genes described in other species to be activated by NRF2 are highlighted in grey.

Furthermore, we created two separate regulatory networks, one related to cytoskeleton and cilia (repressive interactions, Fig. 5A) and one related to redox homeostasis (permissive interactions, Fig. 5B) to better refine and understand the possible co-regulation of several of these targets. The networks show a high level of connectivity and we were able to detect miRNAs that interacted only with NRF2 activated targets (such as ssc-miR-31) (Table 6). Furthermore, we were able to detect miRNAs that seemed to regulate more generally redox homeostasis genes and also miRNAs whose targets came from sets of genes with distinct functions (such as glycolysis, immune system defense, ribosomes, among others), indicating that this response might be related to several other important functions within the cell. We believe that this network can be a powerful tool for analyzing the influence of *M. hyopneumoniae* on the host gene expression in future studies.

**Table 6:** DE genes putatively activated by NRF2 predicted to be targets of DE miRNAs. Expression of miRNAs is reported for intracellular, extracellular and exosome-like vesicles. NS: Not significant; ND: Not detected.

Upregulated gene ID	Downregulated miRNA	miRNA expression		
		Intracellular	Extracellular	Exosome
*AKR1C1*	ssc-miR-101	NS	Downregulated	Downregulated
*AKR1C4*	ssc-miR-31	NS	Downregulated	Downregulated
*AKR1CL1*	ssc-miR-31	NS	Downregulated	Downregulated
*BLVRB*	prediction_13_40037950_40038026_-_3p-353-MC	NS	ND	Downregulated
*EGR1*	antisense-ssc-mir-423_12_44150500_44150579_-_NA-353-MC	NS	NS	Downregulated
*GCLC*	ssc-miR-148b-3p	NS	NS	Downregulated
	ssc-miR-19a	NS	NS	Downregulated
	ssc-miR-19b	NS	NS	Downregulated
*GGT1*	ssc-miR-338	NS	ND	Downregulated
	ssc-miR-140-3p	NS	NS	Downregulated
	ssc-mir-92-shorter/ssc-isomir-92_X_108178486_108178565_-_NA;ssc-mir- 92-shorter/ssc-isomir-92_X_108212408_108212487_-_NA-424-HC	NS	NS	Downregulated
	ssc-miR-92a	NS	NS	Downregulated
	prediction_7_91732076_91732132_+_5p-353-MC	NS	NS	Downregulated
	ssc-miR-31	NS	Downregulated	Downregulated
	antisense-pn8/bta-mir-2320_6_43886629_43886722_-_NA-424-HC	NS	Downregulated	Downregulated
*GSR*	ssc-miR-101	NS	Downregulated	Downregulated
	ssc-miR-22-3p	NS	NS	Downregulated
*HMOX1*	ssc-miR-182	NS	NS	Downregulated
*HSP90AB1*	ssc-miR-101	NS	Downregulated	Downregulated
*NQO1*	ssc-miR-34a	NS	NS	Downregulated
*PGD*	ssc-miR-769-3p	NS	Downregulated	NS
*PIR*	ssc-miR-378	NS	NS	Downregulated
*PRDX1*	ssc-miR-101	NS	Downregulated	Downregulated
*PRDX6*	ssc-miR-10a-5p	NS	NS	Downregulated
	ssc-miR-10b	NS	NS	Downregulated
	ssc-miR-338	NS	ND	Downregulated
*SLC5A10*	ssc-miR-1307	NS	ND	Downregulated
	antisense-pn8/bta-mir-2320_6_43886629_43886722_-_NA-424-HC	NS	Downregulated	Downregulated
*SLC5A6*	ssc-miR-10b	NS	NS	Downregulated
	ssc-miR-338	NS	ND	Downregulated
	ssc-miR-423-3p	NS	ND	Downregulated
	ssc-miR-27b-3p	NS	NS	Downregulated
	prediction_7_91732076_91732132_+_5p-353-MC	NS	NS	Downregulated
	ssc-miR-340	NS	ND	Downregulated
*SOD2*	prediction_7_91732076_91732132_+_5p-353-MC	NS	NS	Downregulated
	ssc-miR-184	Downregulated	ND	ND
	ssc-miR-28-3p	NS	NS	Downregulated
*TKT*	antisense-ssc-mir-423_12_44150500_44150579_-_NA-353-MC	NS	NS	Downregulated
*TXNRD1*	ssc-miR-103	NS	NS	Downregulated
	ssc-miR-182	NS	NS	Downregulated
	ssc-mir-107-shorter/ssc-isomir-107_14_106321702_106321788_-_NA-424-HC	NS	Downregulated	Downregulated
	ssc-miR-107	NS	Downregulated	Downregulated
	ssc-miR-31	NS	Downregulated	Downregulated
*UGT1A6*	ssc-miR-28-3p	NS	NS	Downregulated
	antisense-ssc-mir-103_16_52667657_52667738_+_NA-424-HC	NS	NS	Downregulated
	ssc-miR-769-3p	NS	Downregulated	NS

#### Permissive interactions were enriched in genes regulated by NRF2

Analyzing more in detail the permissive interactions between mRNAs and miRNAs, we identified several miRNAs targeting NRF2 regulated genes (Table 6). *GGT1*, for instance, is predicted to be targeted by 7 different miRNAs, while ssc-mir-31 is predicted to target 4 different genes induced by NRF2 (*AKR1C4, AKR1CL1, GGT1*, and *TXNRD1*).

It was previously reported that *NRF2* can be regulated independently of KEAP1 by miRNAs in human breast cancer cells^83^. Our miRNA target analysis predicted *NRF2* as target of three miRNAs down-regulated in exosome-like vesicles: ssc-mir-340, ssc-mir-19a, and ssc-mir-19b. The first was also predicted to target the transporter gene *SLC6A6*, while ssc-mir-19a and ssc-mir-19b were predicted to target *GCLC*, necessary for glutathione synthesis. In addition, miR-340 has been previously identified negatively regulating *NRF2* expression in human^84^. Conversely, miR-19a and miR-19b have been reported to be down-regulated in the presence of hydrogen peroxide in rats^85, 86^, linking these miRNAs to a response to oxidative stress.

In the literature, other miRNAs were also shown to negatively regulate the expression of *NRF2*: miR-28^83^, miR-101^87^, miR-92a^88^, miR-27b^89^, and miR-34a^90^. All of them were down-regulated in our data and were predicted by this study to regulate NRF2 activated genes (Table 6). In this way, it seems that during *M. hyopneumoniae* infection, there is a global change in gene expression in an attempt to activate antioxidant genes, in association with the down-regulation of miRNAs that negatively regulate these genes.

#### Repressive interactions were related to cytoskeleton and cilia

As concerns the repressive interactions of miRNAs and mRNAs, we identified within the targets of up-regulated miRNAs an enrichment of genes related to cytoskeleton and cilia (Supplementary Table S11). For instance, the gene *TNS3*, related to cytoskeleton, is predicted as target of 16 up-regulated miRNAs (Supplementary Table S11). In addition, several genes encoding for dyneins were predicted as targets of up-regulated miRNAs, with *DNAL1* being target of 7 different miRNAs. Mutations and down-regulation of genes coding ciliary proteins, especially dyneins, were related to primary ciliary dyskinesia^46, 48, 53, 55, 91^, but had not yet been reported in *M. hyopneumoniae* infection. More investigations on this matter should be carried out, since these results seem to be related to one of the main adverse effects caused by this bacterium: ciliostasis.

#### Most DE miRNAs were detected in exosome-like vesicles

Analyzing the up-regulated genes, we identified that besides antioxidant response and protein synthesis, there is an enrichment in GO terms related to cell-cell communication (Fig. 1). The up-regulation of cell-cell communication is noteworthy considering that most DE miRNAs were detected only in the extracellular and exosome-like samples (Supplementary Table S10). Moreover, most of the down-regulated miRNAs of exosome-like vesicles were DE only in these vesicles, indicating that the cells might be selecting specific populations of miRNAs to be packaged into these structures, which could be seen as a specific message they are trying to communicate to other cells. In the same direction, the increase in the number of proteins secreted in vesicles was reported in NPTr cells infected with *M. hyopneumoniae*^92^, indicating that the communication via exosome-like vesicles is important for swine cells during infection. This corroborates our data, suggesting that *M. hyopneumoniae* induces modification on the protein and RNA composition of NPTr-released vesicles and is likely important in the cross-talks between epithelial cells.

The relationship between cell–cell communication and DE miRNAs released by infected cells becomes especially interesting when considering genes related to the NRF2 pathway: we identified DE miRNAs predicted to regulate such genes only in extracellular and exosome-like vesicle samples, with no difference of expression in intracellular samples (Table 6). This might suggest the existence of a mechanism in which infected cells send signals to neighboring cells in order to prevent the repression of these genes or degradation of their RNA products. This becomes even more important due to the fact that exosomal miRNAs were already shown to regulate the inflammatory response in receptor cells from mice^93^. While miRNAs identified in the extracellular total samples might contain sRNAs that reflect degradation remnants due to the presence of RNAses and mRNAs in the extracellular environment, we believe that this is less likely to happen in exosomes, since these vesicles have a membrane that protects their content from extracellular degradation. As exosomes have an important role in cell communication, we should consider the difference in miRNA expression in these vesicles as relevant and speculate on how they might interfere in the gene regulation of neighboring cells. In addition, as exosomes may also affect other cell types, they may contain miRNAs that target genes that were not detected as DE in our samples.

## Concluding remarks

*M. hyopneumoniae* is considered a pathogen with huge negative impact on swine production. However, apart from studies related to adherence to the host cells, little is known about the relationship between this pathogen and the swine host. In this work, we analyzed the changes that *M. hyopneumoniae* induced in gene and miRNA expression in tracheal epithelial cells. As far as we know, this is the first study to establish a link between gene expression of the swine cells and the most deleterious pathogenic effects of *M. hyopneumoniae*, namely its cytotoxic epithelial damage (possibly via hydrogen peroxide production) and induced ciliostasis. However, we are aware that infection with this species involves a large component of the immune system, which is even said to be responsible for the major tissue damage related to the infection. Thus, the results found here reflect the effects of *M. hyopneumoniae* on epithelial cells, while the general picture of the respiratory tract might present different responses to the infection. In this way, it is important to observe that besides the hydrogen peroxide production by this bacterium, other factors can trigger the activation of the cell antioxidant defense during infection. *M. hyopneumoniae* infection is characterized by the infiltration of a large number of leukocytes in the lung tissue, which produce reactive oxygen and nitrogen species, causing tissue damage^38, 94^. Accordingly, systemic infections with *M. hyopneumoniae* have a great potential of causing oxidative stress, so that the transcription of genes activated by NRF2 seems to be important to fight the infection and maintain cellular homeostasis.

In conclusion, we conducted the first study that analyzes mRNA and miRNA differential expression by NGS in epithelial tracheal cells infected with *M. hyopneumoniae*. Our results bring new insights into the interaction between this bacterium and swine epithelial cells, notably the host cellular response through the activation of genes related to antioxidant response and the repression of cytoskeleton and ciliary genes (possibly related to ciliostasis), and open several perspectives related to the understanding of the pathogenicity of this bacterial species.

## Methods

### Cultivation of NPTr cells

NPTr cells^95^ negatively tested for mycoplasma, purchased from Instituto Zooprofilattico Sperimentale della Lombardia e dell’Emilia Romagna, Brescia, Italy, were grown in Minimum Essential Medium with Earle’s Balanced Salts (MEM/EBSS) and 2 mM L-Glutamine (GE Healthcare) supplemented with 10% (v/v) heat-inactivated fetal bovine serum (FBS) (Gibco) and 1% penicillin streptomycin (GE Healthcare). Cells were cultured at $37\,{^{\circ }}\hbox {C}$ with 5% CO2 in a humid atmosphere in T75 $cm^{2}$ flasks and 6-well tissue culture plates. Sub-passages were made when cells reached 70-80% confluence. To isolate exosome-like vesicles, the culture medium was removed, confluent cells were washed with phosphate-buffered saline (PBS) and kept in medium without FBS and antibiotics (exosome depleted) for 24 h.

### Infection of NPTr cells with *M. hyopneumoniae*

*Mycoplasma hyopneumoniae* strain J (ATCC25934) was cultivated in Friis medium^96^ at $37\,{^{\circ }}\hbox {C}$ for 48 h with gentle agitation in a roller drum. Prior to NPTr infection, *Mycoplasma* cells were pelleted, washed with PBS and resuspended in MEM/EBSS medium. Based on the methodology described by Assunção et al. (2005)^97^, we calculated an MOI of 5 *M. hyopneumoniae* for the immunofluorescence experiments and an MOI of 2 for the RNA extraction experiments. NPTr cells were challenged with *M. hyopneumoniae* for 24 h.

### Immunofluorescence microscopy

NPTr cells were grown in cover slips coated with poly-L-lysine (Sigma-Aldrich) for 48 h and infected (or not, in the control conditions) with *M. hyopneumoniae* for 24 h or 1 h in the case of the NFR2 localization experiment. The cells were washed with 1x PBS three times and fixed with 4% paraformaldehyde for 15 min. After permeabilization with 0.2% Triton X-100 for 10 min and blocking with 4% BSA in PBS for 20 min, the cells were incubated with the primary antibodies (rabbit polyclonal anti-SPAse I of *M. hyopneumoniae*^98^, 1:10,000, and mouse monoclonal anti-Sodium/Potassium ATPase alpha (Invitrogen), 1:20, or rabbit polyclonal anti-NRF2 (Thermo Fisher Scientific), 1:200,) overnight at $4\,{^{\circ }}\hbox {C}$ followed by secondary antibodies (Alexa Fluor488-conjugated anti-rabbit (Invitrogen), 1:1,000, and Alexa Fluor555-conjugated anti-mouse (Invitrogen), 1:1,000) incubation for 1 h in the dark. The cells were washed with PBS and the DNA was stained with 100 nM DAPI and actin was marked with 50 nM phalloidin for 20 min. The slides were mounted with Fluoromount (Sigma-Aldrich). Samples were imaged using an Olympus FluoView 1000 confocal microscope. NRF2 fluorescence was quantified by drawing margin to each individual nucleus and measuring the green fluorescence against the cell background. At least 25 nuclei were quantified in each condition with the ImageJ software^99^. The Corrected Total Fluorescence (CTCF) of each nucleus was calculated with the following formula: CTCF = Integrated density – (Area of the selected nucleus x fluorescence of the background reading). Statistical significance was analyzed by One-way ANOVA followed by Dunnett’s multiple comparison test ($\hbox {p} < 0.05$) in GraphPad Prism 7.0 software.

### Exosome extraction

Exosome-like vesicles were purified from 250 mL (25 F75 $cm^{2}$ flasks) of NPTr conditioned medium (as described by Forterre et al.^17^). Briefly, after 24 hours of incubation, the medium was recovered and cell debris and organelles were eliminated by centrifugation at 2,000 x g for 20 min and at 10,000 x g for 30 min. The resulting supernatant was filtered through a $0.22\,\mu \hbox {m}$ filter in order to remove large particles. Exosome-like vesicles were pelleted by ultracentrifugation at 100,000 x g for 90 min $4\,{^{\circ }}\hbox {C}$ (Beckman-Coulter, Optima MAX-XP ultracentrifuge, MLA-55 rotor). The supernatant was recovered and saved for posterior use (SN) and the exosome sediment was washed with 25 mL of cold PBS, centrifuged at 100,000 x g for 70 min $4 \,{^{\circ }}\hbox {C}$ and resuspended in $50\,\mu \hbox {L}$ PBS. Vesicle extractions were performed in duplicates in both conditions of NPTr cells non-infected and infected with *M. hyopneumoniae*. SN portion was submitted to nucleic acid precipitation with 1/10 volume of 3 M sodium acetate and 3 volumes of 100% cold ethanol at $-20\,{^{\circ }}\hbox {C}$ overnight. After centrifugation, the nucleic acid precipitated was allowed to dry at $60\,{^{\circ }}\hbox {C}$ for 1 hour and was ressuspended in $100\,\mu \hbox {L}$ of ultrapure RNAse free water.

### Sample preparation, RNA extraction and sequencing

For mRNA sequencing, a total of 6 samples were prepared: 3 in a control group (CTL) and 3 in the infected group (INF). For miRNA sequencing, we prepared the samples as follows: total intracellular miRNA (INTRA: 3 CTL vs 3 INF), total extracellular small RNA (EXTRA: 2 CTL vs 2 INF), extracellular exosomal miRNA (EXO: 1 pool CTL vs 1 pool INF) and extracellular miRNA from vesicle-free supernatant (SN: 1 pool CTL vs 1 pool INF: a single library was constructed from a pool of 50 biological replicates). Total RNA for mRNA sequencing and sRNA enriched ($< 200$bp) for sRNA sequencing and miRNA analyses were extracted with mirVana kit (Ambion), according to the manufacturer’s instructions. Total extracellular RNA for sRNA sequencing was directly extracted from the culture NPTr cell supernatant after centrifugation of cell debris. Exosome sRNA was extracted after vesicle purification. RNA quality was assessed with Bioanalyzer 2100 (Agilent Genomics). A total of 6 mRNA libraries were prepared (one for each sample) using TruSeq Stranded Total RNA Sample Preparation kit (Illumina) and a total of 14 small RNA libraries were prepared using TruSeq Small RNA Sample Prep kit (Illumina). After quality control, the sequencing of all libraries was performed by HiSeq2500 platform (Illumina). RNA library preparation and sequencing was performed by the Duke University GCB Sequencing Platform (Durham, USA).

### RT-qPCR of selected differentially expressed mRNAs

For RT-qPCR, total RNA of NPTr cells infected and non-infected with *M. hyopneumoniae* was isolated with TRizol (Invitrogen) according to the manufacturer’s instructions. A first-strand cDNA synthesis reaction was conducted by adding $1\,\mu \hbox {g}$ of total RNA to 500 ng of oligo(dT)15 primer (Promega) and 10 mM deoxynucleotide triphosphates. The mixture was heated for $65\,{^{\circ }}\hbox {C}$ for 5 min and then incubated on ice for 5 min. First-strand buffer (Invitrogen), 0.1 M dithiothreitol and 200 U M-MLV RT (Moloney Murine Leukemia Virus Reverse Transcriptase – Invitrogen) were then added to a total volume of $20\,\mu \hbox {L}$. The reaction was incubated at $25\,{^{\circ }}\hbox {C}$ for 10 min and at $37\,{^{\circ }}\hbox {C}$ for 50 min followed by 15 min at $70\,{^{\circ }}\hbox {C}$ for enzyme inactivation. Quantitative PCR (qPCR) assay was performed using 1:5 cDNA as template and Platinum SYBR Green qPCR SuperMix-UDG (Invitrogen) with specific primers (Supplementary Table S13) on 7500 Real-Time PCR Systems (Applied Biosystems). The qPCR reactions were carried out at $90\,{^{\circ }}\hbox {C}$ for 2 min and $95\,{^{\circ }}\hbox {C}$ for 10 min followed by 40 cycles of $95\,{^{\circ }}\hbox {C}$ for 15 s and $60\,{^{\circ }}\hbox {C}$ for 1 min. A melting curve analysis was done to verify the specificity of the synthesized products and the amplification efficiency for each primer was calculated with the LinRegPCR software application^100^. The mRNA levels were normalized against porcine DNA topoisomerase II beta (*TOP2B*) and the relative expression of mRNA was calculated by $2^{-\Delta \Delta {\mathrm{CT}}}$ method. The threshold cycle (CT) of each target test represents the average of three reactions. Three independent biological replicates were done for each target gene. Statistical analyses were performed using the GraphPad Prism 7.0 software with a two-tailed unpaired t-test to compare the relative expression between infected and non-infected NPTr cells ($\hbox {p} < 0.05$).

### Computational analysis of sequencing data

#### Preprocessing of raw reads

The reads from sRNA-seq and total RNA-seq were processed separately. The raw reads were filtered for low quality, and adapter sequences were trimmed with CUTADAPT^101^. Total mRNA-seq clean reads were mapped against the porcine reference genome from Ensembl (Sscrofa10.2) with STAR^102^ and the raw counts of genes were obtained with HTSeq^103^. Only uniquely mapped reads were used for further analyses.

For miRNA prediction, clean sRNA-seq reads were mapped against the porcine genome with Bowtie^104^. We used intracellular samples as input for miRDeep2^76^ and kept predictions that had a score of at least 5. After this, we collapsed similar predictions and obtained a total of 773 clusters (773 miRNAs), of which 478 were novel miRNAs. We created a porcine miRNA DB (ssc-miRNA-DB), by including the 411 annotated porcine miRNAs in version 21 of miRBase^77, 105^ along with the 722 miRNAs characterized by Martini et al. (2014)^78^ and our novel predictions. Reads from all samples were mapped against this database and a matrix of counts was generated in order to identify DE miRNAs.

#### Differential expression of mRNA data

Raw counts were used as input for DE gene analysis. We detected possible DE genes in both EdgeR^106^ and DESeq2^107^ packages in R. DESeq2 was run for genes that had a total count of at least 10 in all libraries, with the method’s default normalization. EdgeR was used with TMM normalization and general linear model fit, only for genes with cpm greater than 1 in at least 2 libraries. After testing, the adjusted p-values (p-adj) for both methods were adjusted with the Benjamini-Hochberg^108^ correction for multitesting. Genes were considered DE when p-adj $< 0.05$ and genes with the most pronounced logFC were selected individually for further investigation. Overall, DESeq2 detected more significant p-adj with less accentuated LogFC, while EdgeR detected less significant false discovery rates (FDR) with more extreme LogFCs. Both techniques have been widely used separately and together in several publications, and in order to select good candidates for testing, we took into account the results of both methods. For GO functional analyses, we also used the complete list of DE genes whenever LogFC was greater than 0.1 (up-regulated) or smaller than $-0.1$ (down-regulated). The complete lists from each method were used separately, and we compared the overall outcomes to check the robustness of our results.

#### Differential expression of miRNA data

The same pipeline used for DE mRNA was performed for total intracellular and total extracellular miRNAs, since we had biological replicates in both cases. In the case where we had no replicates (instead, a single library was constructed from a pool of 50 biological replicates), we used GFOLD^109^ which provides a generalized fold change for ranking DE genes. GFOLD is said to overcome the shortcomings of p-value and fold change of the existing methods and can provide a more stable and biological meaningful gene ranking when a single biological replicate is available. In this case, we selected miRNAs with GFOLD $> 2$ or $< -2$ for functional analyses.

#### miRNA target prediction

DE miRNAs were used as input to detect putative interactions with the UTRs of Ensembl transcripts in the porcine genome. We used three methods to detect target pairs: miRanda^110^, TargetScan^111^ and PITA^112^, and one method to validate the hybridization of a target pair, RNAhybrid^113^. We kept only targets that were predicted by at least two distinct tools and used the following thresholds: score in miRanda $> 140$, DDG from PITA $< -5$, score in RNAhybrid $< -15$. Since TargetScan does not provide a continuous scoring system, we only validated whenever there was a prediction of at least 6mers. Based on these, we chose from the target list only genes that were detected as DE in this study, and subsequentially we only considered target pairs of miRNA-mRNA that had inversed LogFC expression.

#### Functional analysis of DE mRNAs and targets of DE miRNAs

Functional analysis took into account as input either the list of DE mRNAs itself or the list of targets predicted for the DE miRNAs. We performed a GO enrichment analysis^114^ to find out which functions were over or underrepresented in each gene list. P-values for enriched GO terms were adjusted with the Benjamini-Hochberg^108^ correction for multitesting. GO terms and pathways with p-adj $< 0.05$ were defined as significantly enriched. The GO terms were reduced to representative non redundant terms with the use of the REVIGO tool^115^.

#### Regulatory network reconstruction and analysis

We created a general regulatory network of the host response to the bacterial infection with the DE miRNAs and target mRNAs detected in this study (Supplementary Fig. S9 and Supplementary File S1). In this network, we also included information about interactions from the BioGRID v3.4 database^80, 81^, a general repository that includes experimentally validated physical and genetic interactions. We used human-based official gene symbols to include information from BioGRID, Cytoscape^116^ to draw the networks, and the ClueGO plugin^82^ to perform functional enrichment analysis. We further manually curated the networks for genes and miRNAs related with redox homeostasis (permissive regulatory network), as well as cytoskeleton and cilia (repressive regulatory network).

## Supplementary Information

Supplementary material 1

Supplementary material 2

Supplementary material 3

## Data Availability

All sequences were submitted to the Sequence Read Archive from NCBI: SRA accession: PRJNA545822.

## References

[1] Maes D, Verdonck M, Deluyker H, de Kruif A (1996). Enzootic pneumonia in pigs. Vet. Q..

[2] Maes D (2018). Update on Mycoplasma hyopneumoniae infections in pigs: knowledge gaps for improved disease control. Transbound. Emerg. Dis..

[3] Djordjevic SP, Cordwell SJ, Djordjevic MA, Wilton J, Minion FC (2004). Proteolytic processing of the *Mycoplasma hyopneumoniae* cilium adhesin. Infect. Immun..

[4] DeBey MC, Ross RF (1994). Ciliostasis and loss of cilia induced by *Mycoplasma hyopneumoniae* in porcine tracheal organ cultures. Infect. Immun..

[5] Ferrarini MG (2018). Hydrogen peroxide production and myo-inositol metabolism as important traits for virulence of Mycoplasma hyopneumoniae. Mol. Microbiol..

[6] Bartel DP (2004). Micrornas: genomics, biogenesis, mechanism, and function. Cell.

[7] Luo Y (2017). Detection of dietetically absorbed maize-derived micrornas in pigs. Sci. Rep..

[8] Lawless, N., Foroushani, A. B., McCabe, M. S., O’Farrelly, C. & Lynn, D. J. Next generation sequencing reveals the expression of a unique mirna profile in response to a gram-positive bacterial infection. *PLoS ONE* **8**, e57543 (2013). 10.1371/journal.pone.0057543 PMC3589390 23472090

[9] Duval, M., Cossart, P. & Lebreton, A. Mammalian micrornas and long noncoding rnas in the host-bacterial pathogen crosstalk. In *Seminars in cell & developmental biology*, vol. 65, 11–19 (Elsevier, 2017). 10.1016/j.semcdb.2016.06.016 PMC7089780 27381344

[10] Lim LP (2005). Microarray analysis shows that some micrornas downregulate large numbers of target mrnas. Nature.

[11] Kim VN, Nam J-W (2006). Genomics of microrna. Trends Genet..

[12] Valadi H (2007). Exosome-mediated transfer of mrnas and micrornas is a novel mechanism of genetic exchange between cells. Nat. Cell Biol..

[13] Rome S (2013). Are extracellular micrornas involved in type 2 diabetes and related pathologies?. Clin. Biochem..

[14] Chen L (2014). Epigenetic regulation of connective tissue growth factor by microrna-214 delivery in exosomes from mouse or human hepatic stellate cells. Hepatology.

[15] Schorey JS, Cheng Y, Singh PP, Smith VL (2015). Exosomes and other extracellular vesicles in host–pathogen interactions. EMBO Rep..

[16] Rana S, Yue S, Stadel D, Zöller M (2012). Toward tailored exosomes: the exosomal tetraspanin web contributes to target cell selection. Int. J. Biochem. Cell Biol..

[17] Forterre A (2014). Myotube-derived exosomal mirnas downregulate sirtuin1 in myoblasts during muscle cell differentiation. Cell Cycle.

[18] Silverman JM, Reiner NE (2011). Exosomes and other microvesicles in infection biology: organelles with unanticipated phenotypes. Cell. Microbiol..

[19] Muxel SM, Laranjeira-Silva MF, Zampieri RA, Floeter-Winter LM (2017). Leishmania (Leishmania) amazonensis induces macrophage mir-294 and mir-721 expression and modulates infection by targeting nos2 and l-arginine metabolism. Sci. Rep..

[20] Bao H (2015). Genome-wide whole blood micrornaome and transcriptome analyses reveal mirna-mrna regulated host response to foodborne pathogen Salmonella infection in swine. Sci. Rep..

[21] Staedel C, Darfeuille F (2013). Micro rna s and bacterial infection. Cell. Microbiol..

[22] Maudet C, Mano M, Eulalio A (2014). Micrornas in the interaction between host and bacterial pathogens. FEBS Lett..

[23] Zielinski G, Ross R (1990). Effect of growth in cell cultures and strain on virulence of *Mycoplasma hyopneumoniae* for swine. Am. J. Vet. Res..

[24] Young TF, Thacker EL, Erickson BZ, Ross RF (2000). A tissue culture system to study respiratory ciliary epithelial adherence of selected swine mycoplasmas. Vet. Microbiol..

[25] Burnett TA (2006). P159 is a proteolytically processed, surface adhesin of *Mycoplasma hyopneumoniae*: defined domains of p159 bind heparin and promote adherence to eukaryote cells. Mol. Microbiol..

[26] Cho H-Y, Reddy SP, Kleeberger SR (2006). Nrf2 defends the lung from oxidative stress. Antioxid. Redox Signal..

[27] Ma Q (2013). Role of nrf2 in oxidative stress and toxicity. Annu. Rev. Pharmacol. Toxicol..

[28] Thimmulappa RK (2016). Nrf2 is a critical regulator of the innate immune response and survival during experimental sepsis. J. Clin. Investig..

[29] Athale J (2012). Nrf2 promotes alveolar mitochondrial biogenesis and resolution of lung injury in staphylococcus aureus pneumonia in mice. Free Radical Biol. Med..

[30] Reddy NM (2009). Innate immunity against bacterial infection following hyperoxia exposure is impaired in nrf2-deficient mice. J. Immunol..

[31] Gomez JC, Dang H, Martin JR, Doerschuk CM (2016). Nrf2 modulates host defense during *Streptococcus pneumoniae* pneumonia in mice. J. Immunol..

[32] Kensler TW, Wakabayashi N, Biswal S (2007). Cell survival responses to environmental stresses via the keap1-nrf2-are pathway. Annu. Rev. Pharmacol. Toxicol..

[33] Fourquet S, Guerois R, Biard D, Toledano MB (2010). Activation of nrf2 by nitrosative agents and h2o2 involves keap1 disulfide formation. J. Biol. Chem..

[34] Hames C, Halbedel S, Hoppert M, Frey J, Stülke J (2009). Glycerol metabolism is important for cytotoxicity of *Mycoplasma pneumoniae*. J. Bacteriol..

[35] Vilei EM, Frey J (2001). Genetic and biochemical characterization of glycerol uptake in *Mycoplasma mycoides* subsp. *mycoides *sc: Its impact on h2o2production and virulence. Clin. Diagn. Lab. Immunol..

[36] Bischof DF, Janis C, Vilei EM, Bertoni G, Frey J (2008). Cytotoxicity of mycoplasma mycoides subsp. mycoides small colony type to bovine epithelial cells. Infect. Immun..

[37] Halbedel S, Hames C, Stülke J (2004). In vivo activity of enzymatic and regulatory components of the phosphoenolpyruvate: sugar phosphotransferase system in mycoplasma pneumoniae. J. Bacteriol..

[38] Biswal S, Thimmulappa RK, Harvey CJ (2012). Experimental therapeutics of nrf2 as a target for prevention of bacterial exacerbations in copd. Proc. Am. Thorac. Soc..

[39] Nioi P, McMahon M, Itoh K, Yamamoto M, Hayes JD (2003). Identification of a novel nrf2-regulated antioxidant response element (are) in the mouse nad (p) h: quinone oxidoreductase 1 gene: reassessment of the are consensus sequence. Biochem. J..

[40] Rice, P., Longden, I. & Bleasby, A. Emboss: the European molecular biology open software suite (2000). 10.1016/s0168-9525(00)02024-2 10827456

[41] Araake M (1982). Comparison of ciliostasis by mycoplasmas in mouse and chicken tracheal organ cultures. Microbiol. Immunol..

[42] Abu-Zahr M, Butler M (1976). Growth, cytopathogenicity and morphology of *Mycoplasma gallisepticum* and *M. gallinarum* in tracheal explants. J. Comp. Pathol..

[43] Chaudhry R, Ghosh A, Chandolia A (2016). Pathogenesis of Mycoplasma pneumoniae: an update. Indian J. Med. Microbiol..

[44] Matsuyama M (2018). Transcriptional response of respiratory epithelium to nontuberculous mycobacteria. Am. J. Respir. Cell Mol. Biol..

[45] Kobayashi D, Takeda H (2012). Ciliary motility: the components and cytoplasmic preassembly mechanisms of the axonemal dyneins. Differentiation.

[46] Tarkar A (2013). Dyx1c1 is required for axonemal dynein assembly and ciliary motility. Nat. Genet..

[47] Wang W-J (2013). Cep162 is an axoneme-recognition protein promoting ciliary transition zone assembly at the cilia base. Nat. Cell Biol..

[48] Schueler M (2015). Dcdc2 mutations cause a renal-hepatic ciliopathy by disrupting wnt signaling. Am. J. Human Genet..

[49] May-Simera HL (2016). Loss of macf1 abolishes ciliogenesis and disrupts apicobasal polarity establishment in the retina. Cell Rep..

[50] Shi L, Koll F, Arnaiz O, Cohen J (2018). The ciliary protein ift 57 in the macronucleus of paramecium. J. Eukaryot. Microbiol..

[51] Yasunaga T (2015). The polarity protein inturned links nphp4 to daam1 to control the subapical actin network in multiciliated cells. J. Cell Biol..

[52] Hegan PS, Ostertag E, Geurts AM, Mooseker MS (2015). Myosin id is required for planar cell polarity in ciliated tracheal and ependymal epithelial cells. Cytoskeleton.

[53] Fernandez-Gonzalez A, Kourembanas S, Wyatt TA, Mitsialis SA (2009). Mutation of murine adenylate kinase 7 underlies a primary ciliary dyskinesia phenotype. Am. J. Respir. Cell Mol. Biol..

[54] Zhou J, Yang F, Leu NA, Wang PJ (2012). Mns1 is essential for spermiogenesis and motile ciliary functions in mice. PLoS Genet..

[55] Inaba Y (2016). Transport of the outer dynein arm complex to cilia requires a cytoplasmic protein lrrc6. Genes Cells.

[56] Arachchige, S. N. K. *et al.* Differential response of the chicken trachea to chronic infection with virulent Mycoplasma gallisepticum strain ap3as and vaxsafe mg (strain ts-304): a transcriptional profile. *Infect. Immun.* **88**, (2020). 10.1128/IAI.00053-20 PMC7171234 32122943

[57] Girón JA, Lange M, Baseman JB (1996). Adherence, fibronectin binding, and induction of cytoskeleton reorganization in cultured human cells by Mycoplasma penetrans. Infect. Immun..

[58] Raymond B (2018). Mycoplasma hyopneumoniae resides intracellularly within porcine epithelial cells. Sci. Rep..

[59] Mirvis M, Stearns T, Nelson WJ (2018). Cilium structure, assembly, and disassembly regulated by the cytoskeleton. Biochem. J..

[60] Raymond B (2018). Extracellular actin is a receptor for *Mycoplasma hyopneumoniae*. Front. Cell. Infect. Microbiol..

[61] Kang M-I, Kobayashi A, Wakabayashi N, Kim S-G, Yamamoto M (2004). Scaffolding of keap1 to the actin cytoskeleton controls the function of nrf2 as key regulator of cytoprotective phase 2 genes. Proc. Nat. Acad. Sci..

[62] Mack GJ, Compton DA (2001). Analysis of mitotic microtubule-associated proteins using mass spectrometry identifies astrin, a spindle-associated protein. Proc. Nat. Acad. Sci..

[63] Kim YH, Choi JY, Jeong Y, Wolgemuth DJ, Rhee K (2002). Nek2 localizes to multiple sites in mitotic cells, suggesting its involvement in multiple cellular functions during the cell cycle. Biochem. Biophys. Res. Commun..

[64] Woolner, S., O’Brien, L. L., Wiese, C. & Bement, W. M. Myosin-10 and actin filaments are essential for mitotic spindle function. *J. Cell Biol.* **182**, 77–88 (2008). 10.1083/jcb.200804062 PMC2447898 18606852

[65] Bolanos-Garcia VM, Blundell TL (2011). Bub1 and bubr1: multifaceted kinases of the cell cycle. Trends Biochem. Sci..

[66] Wolter P (2012). Gas2l3, a target gene of the dream complex, is required for proper cytokinesis and genomic stability. J. Cell Sci..

[67] Xu X-L (2012). The microtubule-associated protein aspm regulates spindle assembly and meiotic progression in mouse oocytes. PLoS ONE.

[68] Matson DR, Stukenberg PT (2014). Cenp-i and aurora b act as a molecular switch that ties rzz/mad1 recruitment to kinetochore attachment status. J. Cell Biol..

[69] Wadsworth P (2015). Tpx2. Curr. Biol..

[70] Cho RJ (2001). Transcriptional regulation and function during the human cell cycle. Nat. Genet..

[71] Shirin H (1999). Helicobacter pylori inhibits the g1 to s transition in ags gastric epithelial cells. Cancer Res..

[72] Marchès O (2003). Enteropathogenic and enterohaemorrhagic Escherichia coli deliver a novel effector called cif, which blocks cell cycle g2/m transition. Mol. Microbiol..

[73] Jones A, Jonsson A-B, Aro H (2007). Neisseria gonorrhoeae infection causes a g1 arrest in human epithelial cells. FASEB J..

[74] Alekseeva L (2013). Staphylococcus aureus-induced g2/m phase transition delay in host epithelial cells increases bacterial infective efficiency. PLoS ONE.

[75] Siqueira FM (2016). Mycoplasma non-coding rna: identification of small rnas and targets. BMC Genom..

[76] Friedländer MR, Mackowiak SD, Li N, Chen W, Rajewsky N (2011). mirdeep2 accurately identifies known and hundreds of novel microrna genes in seven animal clades. Nucl. Acids Res..

[77] Kozomara A, Griffiths-Jones S (2013). mirbase: annotating high confidence micrornas using deep sequencing data. Nucl. Acids Res..

[78] Martini P (2014). Tissue-specific expression and regulatory networks of pig micrornaome. PLoS ONE.

[79] Bartel DP (2018). Metazoan micrornas. Cell.

[80] Stark C (2006). Biogrid: a general repository for interaction datasets. Nucl. Acids Res..

[81] Chatr-Aryamontri A (2017). The biogrid interaction database: 2017 update. Nucl. Acids Res..

[82] Bindea G (2009). Cluego: a cytoscape plug-in to decipher functionally grouped gene ontology and pathway annotation networks. Bioinformatics.

[83] Yang M, Yao Y, Eades G, Zhang Y, Zhou Q (2011). Mir-28 regulates nrf2 expression through a keap1-independent mechanism. Breast Cancer Res. Treat..

[84] Shi L (2015). mir-340 reverses cisplatin resistance of hepatocellular carcinoma cell lines by targeting nrf2-dependent antioxidant pathway. Asian Pac. J. Cancer Prev..

[85] Xu J (2016). mir-19b attenuates h2o2-induced apoptosis in rat h9c2 cardiomyocytes via targeting pten. Oncotarget.

[86] Hong J (2017). Transcriptional downregulation of microrna-19a by ros production and nf-$$\kappa $$b deactivation governs resistance to oxidative stress-initiated apoptosis. Oncotarget.

[87] Gao A-M, Zhang X-Y, Ke Z-P (2017). Apigenin sensitizes bel-7402/adm cells to doxorubicin through inhibiting mir-101/nrf2 pathway. Oncotarget.

[88] Liu H (2017). Regulation of mir-92a on vascular endothelial aging via mediating nrf2-keap1-are signal pathway. Eur. Rev. Med. Pharmacol. Sci..

[89] Yang H (2015). Activation of a novel c-myc-mir27-prohibitin 1 circuitry in cholestatic liver injury inhibits glutathione synthesis in mice. Antioxid. Redox Signal..

[90] Alural B, Ozerdem A, Allmer J, Genc K, Genc S (2015). Lithium protects against paraquat neurotoxicity by nrf2 activation and mir-34a inhibition in sh-sy5y cells. Front. Cell. Neurosci..

[91] Milara J, Armengot M, Mata M, Morcillo EJ, Cortijo J (2010). Role of adenylate kinase type 7 expression on cilia motility: possible link in primary ciliary dyskinesia. Am. J. Rhinol. Allergy.

[92] Zimmer FMdAL, Paludo GP, Moura H, Barr JR, Ferreira HB (2019). Differential secretome profiling of a swine tracheal cell line infected with mycoplasmas of the swine respiratory tract. J. Proteom..

[93] Alexander M (2015). Exosome-delivered micrornas modulate the inflammatory response to endotoxin. Nat. Commun..

[94] Muneta Y (2008). Immune response of gnotobiotic piglets against *Mycoplasma hyopneumoniae*. J. Vet. Med. Sci..

[95] Ferrari M (2003). Establishment and characterization of two new pig cell lines for use in virological diagnostic laboratories. J. Virol. Methods.

[96] Friis N (1975). Some recommendations concerning primary isolation of *Mycoplasma suipneumoniae* and *Mycoplasma flocculare* a survey. Nordisk veterinaermedicin.

[97] Assunção P (2005). Evaluation of *Mycoplasma hyopneumoniae* growth by flow cytometry. J. Appl. Microbiol..

[98] Moitinho-Silva L (2012). *Mycoplasma hyopneumoniae* type i signal peptidase: expression and evaluation of its diagnostic potential. Vet. Microbiol..

[99] Schneider CA, Rasband WS, Eliceiri KW (2012). Nih image to imagej: 25 years of image analysis. Nat. Methods.

[100] Ruijter J (2009). Amplification efficiency: linking baseline and bias in the analysis of quantitative pcr data. Nucl. Acids Res..

[101] Martin M (2011). Cutadapt removes adapter sequences from high-throughput sequencing reads. EMBnet. J..

[102] Dobin A (2013). Star: ultrafast universal rna-seq aligner. Bioinformatics.

[103] Anders S, Pyl PT, Huber W (2015). Htseq—a python framework to work with high-throughput sequencing data. Bioinformatics.

[104] Langmead B, Trapnell C, Pop M, Salzberg SL (2009). Ultrafast and memory-efficient alignment of short dna sequences to the human genome. Genome Biol..

[105] Griffiths-Jones S (2004). The microrna registry. Nucl. Acids Res..

[106] Robinson MD, McCarthy DJ, Smyth GK (2010). edger: a bioconductor package for differential expression analysis of digital gene expression data. Bioinformatics.

[107] Love MI, Huber W, Anders S (2014). Moderated estimation of fold change and dispersion for rna-seq data with deseq2. Genome Biol..

[108] Benjamini Y, Hochberg Y (1995). Controlling the false discovery rate: a practical and powerful approach to multiple testing. J. R. Stat. Soc.: Ser. B (Methodol.).

[109] Feng J (2012). Gfold: a generalized fold change for ranking differentially expressed genes from rna-seq data. Bioinformatics.

[110] Betel D, Koppal A, Agius P, Sander C, Leslie C (2010). Comprehensive modeling of microrna targets predicts functional non-conserved and non-canonical sites. Genome Biol..

[111] Lewis BP, Shih I-H, Jones-Rhoades MW, Bartel DP, Burge CB (2003). Prediction of mammalian microrna targets. Cell.

[112] Kertesz M, Iovino N, Unnerstall U, Gaul U, Segal E (2007). The role of site accessibility in microrna target recognition. Nat. Genet..

[113] Krüger J, Rehmsmeier M (2006). Rnahybrid: microrna target prediction easy, fast and flexible. Nucl. Acids Res..

[114] Ashburner M (2000). Gene ontology: tool for the unification of biology. Nat. Genet..

[115] Supek F, Bošnjak M, Škunca N, Šmuc T (2011). Revigo summarizes and visualizes long lists of gene ontology terms. PLoS ONE.

[116] Shannon P (2003). Cytoscape: a software environment for integrated models of biomolecular interaction networks. Genome Res..

[117] Hayes JD, McMahon M (2009). Nrf2 and keap1 mutations: permanent activation of an adaptive response in cancer. Trends Biochem. Sci..

[118] Hayes JD, Dinkova-Kostova AT (2014). The nrf2 regulatory network provides an interface between redox and intermediary metabolism. Trends Biochem. Sci..

[119] Brzóska K, Stępkowski TM, Kruszewski M (2014). Basal pir expression in hela cells is driven by nrf2 via evolutionary conserved antioxidant response element. Mol. Cell. Biochem..

[120] Li D, Ma S, Ellis EM (2015). Nrf2-mediated adaptive response to methyl glyoxal in hepg2 cells involves the induction of akr7a2. Chem. Biol. Interact..

[121] Graham DB (2018). Nitric oxide engages an anti-inflammatory feedback loop mediated by peroxiredoxin 5 in phagocytes. Cell Rep..

[122] Reszka E (2013). Expression of nrf2 and nrf2-modulated genes in peripheral blood leukocytes of bladder cancer males. Neoplasma.

[123] Dai L-L, Gao J-X, Zou C-G, Ma Y-C, Zhang K-Q (2015). mir-233 modulates the unfolded protein response in *C. elegans* during *Pseudomonas aeruginosa* infection. PLoS Pathog..

[124] Derrick T (2013). Conjunctival microrna expression in inflammatory trachomatous scarring. PLoS Negl. Trop. Dis..

[125] Derrick T (2015). Inverse relationship between microrna-155 and-184 expression with increasing conjunctival inflammation during ocular Chlamydia trachomatis infection. BMC Infect. Dis..

[126] Liang G (2016). Altered microrna expression and pre-mrna splicing events reveal new mechanisms associated with early stage Mycobacterium avium subspecies paratuberculosis infection. Sci. Rep..

[127] Siddle KJ (2015). Bacterial infection drives the expression dynamics of micrornas and their isomirs. PLoS Genet..

[128] Naqvi AR, Fordham JB, Nares S (2015). mir-24, mir-30b, and mir-142-3p regulate phagocytosis in myeloid inflammatory cells. J. Immunol..

[129] Liu Z (2010). Up-regulated microrna-146a negatively modulate *Helicobacter pylori*-induced inflammatory response in human gastric epithelial cells. Microb. Infect..

[130] Li N (2012). *H. pylori* related proinflammatory cytokines contribute to the induction of mir-146a in human gastric epithelial cells. Mol. Biol. Rep..

[131] Liu Z (2013). Microrna-146a negatively regulates ptgs2 expression induced by helicobacter pylori in human gastric epithelial cells. J. Gastroenterol..

[132] Cheng Y (2012). Downregulation of mir-27a* and mir-532-5p and upregulation of mir-146a and mir-155 in lps-induced raw264. 7 macrophage cells. Inflammation.

[133] Derrick, T. *et al.* mirnas that associate with conjunctival inflammation and ocular *Chlamydia trachomatis* infection do not predict progressive disease. *Pathog. Dis.* **75**, (2017). 10.1093/femspd/ftx016 PMC5399916 28175294

[134] Zheng L (2015). Differential microrna expression in human macrophages with *Mycobacterium tuberculosis* infection of beijing/w and non-beijing/w strain types. PLoS ONE.

[135] Matsushima K (2011). Microrna signatures in *Helicobacter pylori*-infected gastric mucosa. Int. J. Cancer.

[136] Xue X (2014). Downregulation of micro rna-107 in intestinal cd 11c+ myeloid cells in response to microbiota and proinflammatory cytokines increases il-23p19 expression. Eur. J. Immunol..

[137] Xue F (2013). mir-31 regulates interleukin 2 and kinase suppressor of ras 2 during t cell activation. Genes Immun..

[138] Podolska A (2012). Profiling micrornas in lung tissue from pigs infected with *Actinobacillus pleuropneumoniae*. BMC Genom..

[139] Noto JM (2013). Strain-specific suppression of microrna-320 by carcinogenic *Helicobacter pylori* promotes expression of the antiapoptotic protein mcl-1. Am. J. Physiol.-Gastrointest. Liver Physiol..

