# Supplementary material for: Mycoplasma hyopneumoniae J elicits an antioxidant response and decreases the expression of ciliary genes in infected swine epithelial cells
Source: Sci Rep. 2020 Aug 13;10:13707. doi: 10.1038/s41598-020-70040-y (PMC7426424; doi:10.1038/s41598-020-70040-y)
Supplement: Supplementary file 1 — Supplementary material 1 [file 41598_2020_70040_MOESM1_ESM.pdf]

Supplementary Material: Figures

ARTICLE: *Mycoplasma hyopneumoniae* J elicits an antioxidant response and decreases the expression of ciliary genes in infected swine epithelial cells

Scheila G. Mucha[1+], Mariana G. Ferrarini[1,2+], Carol C. Moraga[2,3], Alex Di Genova[2,3], Laurent Guyon[4], Florence Tardy[5,6], Sophie Rome[7], Marie-France Sagot[2,3]*, Arnaldo Zaha[1]*

[1] Centro de Biotecnologia, Universidade Federal do Rio Grande do Sul, Porto Alegre, Brazil.
[2] Université de Lyon, Université Lyon 1, CNRS, Laboratoire de Biométrie et Biologie Evolutive UMR 5558, F-69622 Villeurbanne, France.
[3] ERABLE, Inria, Lyon, France.
[4] Univ. Grenoble Alpes, CEA, INSERM, IRIG, Biology of Cancer and Infection UMR S 1036, F-38000 Grenoble, France.
[5] Universtité de Lyon, Anses, Laboratoire de Lyon, UMR Mycoplasmoses des Ruminants, Lyon, France.
[6] Université de Lyon, VetAgro Sup, UMR Mycoplasmoses des Ruminants, Lyon, France.
[7] CarMeN Laboratory, INSERM 1060/INRA 1397, Université de Lyon, Faculté de Médecine Lyon-Sud, 69310 Pierre-Bénite, France.

+ These authors contributed equally to this work.
* Corresponding authors

Arnaldo Zaha: zaha@cbiot.ufrgs.br
Marie-France Sagot: marie-france.sagot@inria.fr

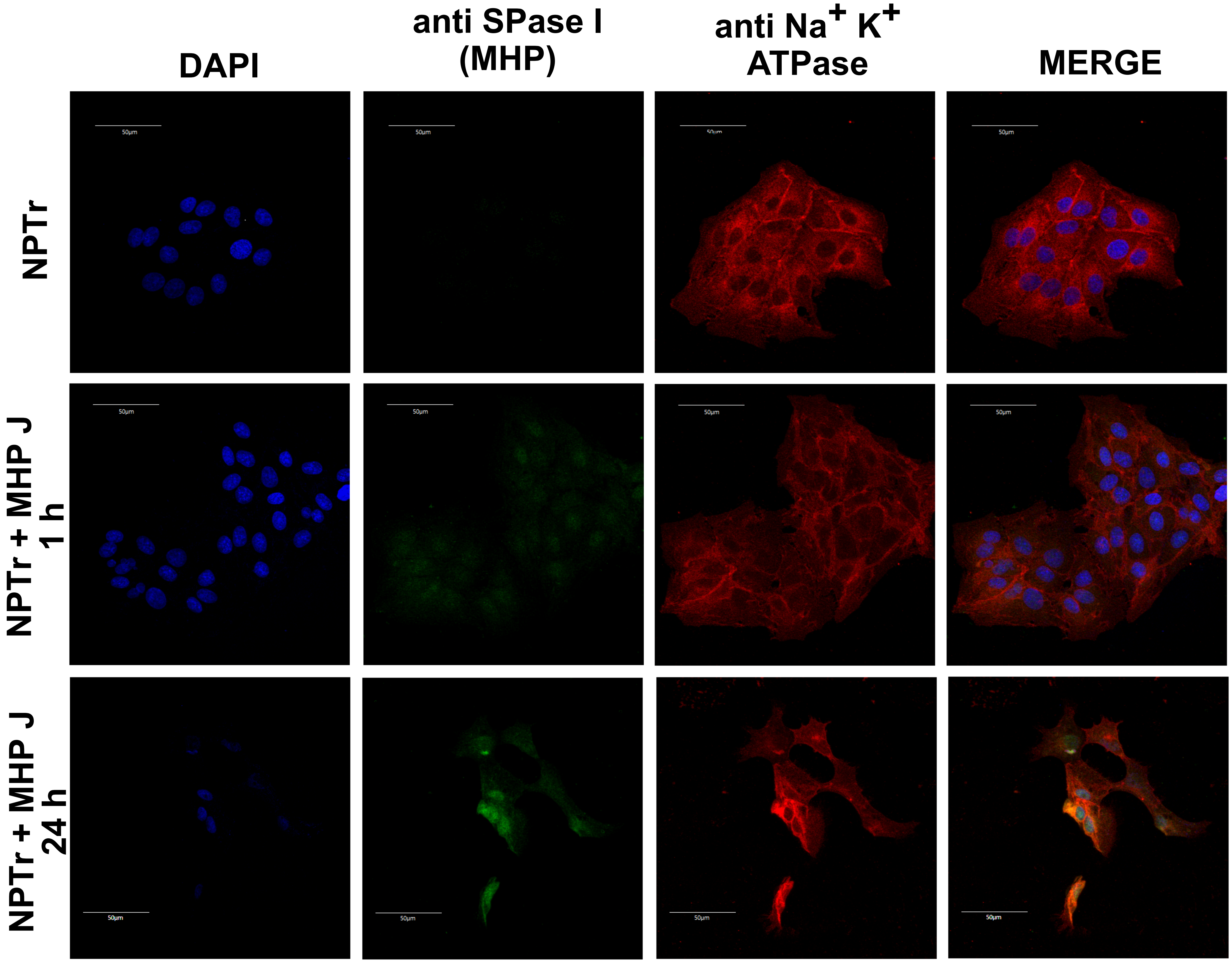

Figure S1. Analysis of *M. hyopneumoniae* adherence to swine epithelial cells 1 h and 24 h post-infection. Results of the immunofluorescence microscopy indicating adherence of *M. hyopneumoniae* to the membrane of the swine cells. After 1 h few mycoplasmas were adhered to the host cells if compared to 24 h. Eukaryotic cell membranes were labeled with mouse anti-Sodium/ Potassium ATPase alpha (red), *M. hyopneumoniae* was detected with rabbit anti-SPAseI (green) and nuclei were stained with DAPI (blue). NPTr - non-infected cells. NPTr+MHP - NPTr cells infected with *M. hyopneumoniae* strain J.

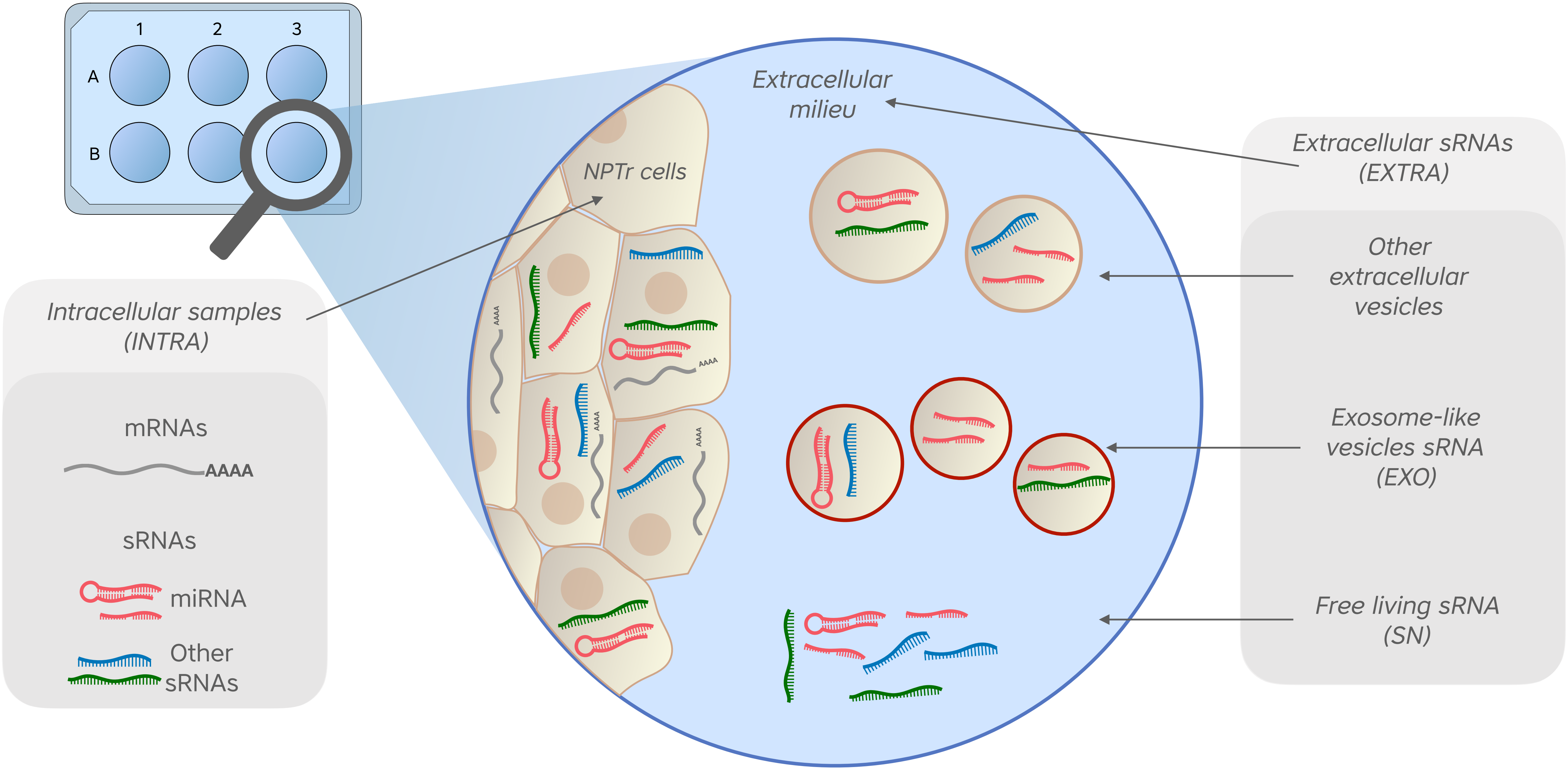

Figure S2. Experimental design of samples. Eukaryotic cells express mRNAs and miRNAs and also export some of these molecules to the extracellular environment in vesicles or in free form. Therefore, besides analyzing differential expression of intracellular mRNA samples (INTRA mRNAs), we also analized DE miRNAs in distinct cell compartments. In this way, we extracted sRNAs from: i) cells (INTRA); ii) from the medium of cultured cells, which contained all kinds of vesicles released by the cells (microvesicles, exosomes, apoptotic bodies, etc) as well as free-living sRNAs (EXTRA); iii) from exosome-like vesicles released by cells (EXO); and iv) from the supernatant of the ultracentrifugation of exosome-like vesicles, which contained free-living miRNAs (SN). The sRNAs of all these sources were sequenced and the differential expression between infected and non infected samples was analyzed.

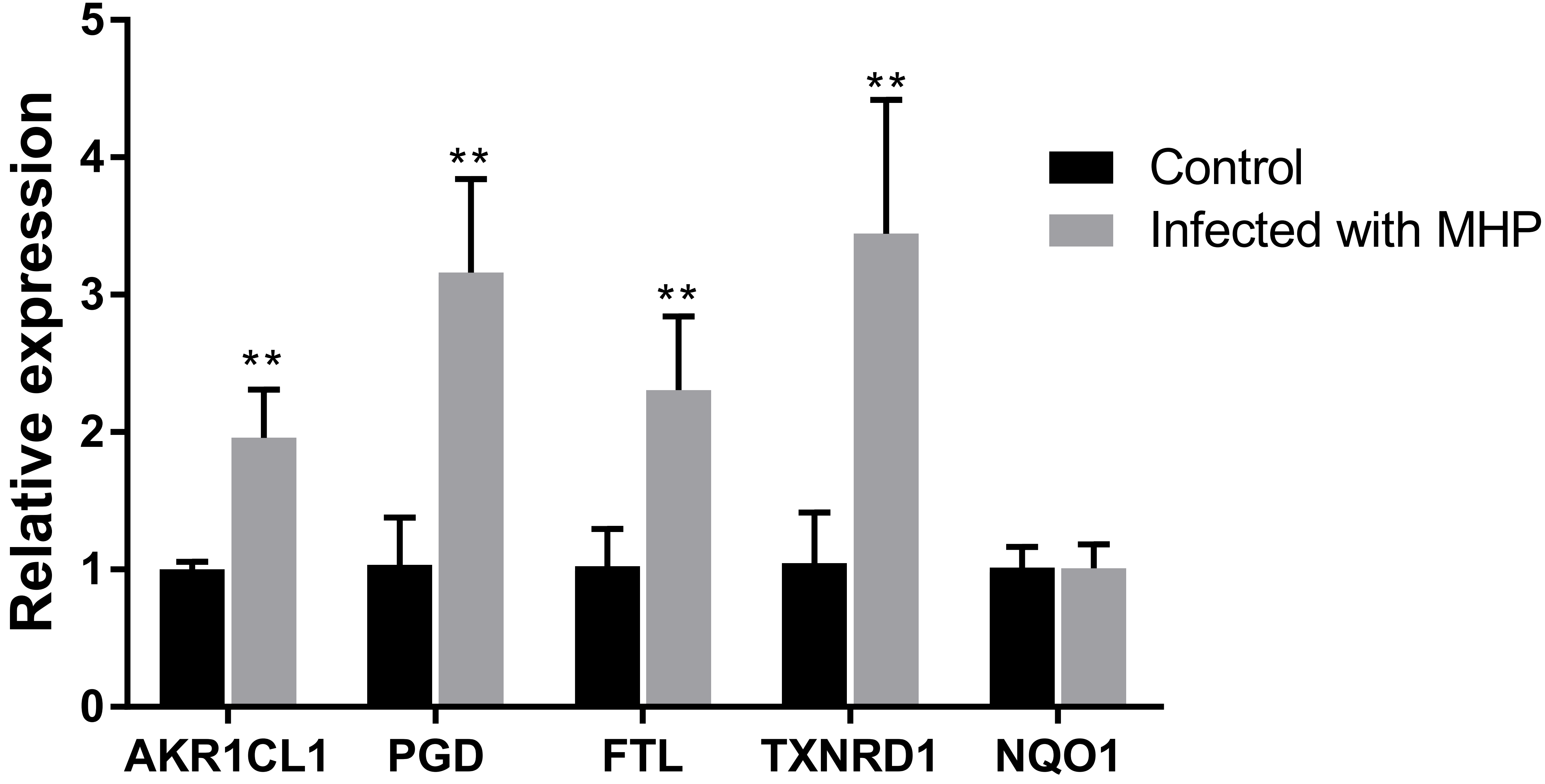

Figure S3. RT-qPCR of selected NRF2 targets. Up-regulated genes described to be activated by the transcription factor NRF2 were selected for experimental validation by RT-qPCR. Of these, four of them (*AKR1CL1*, *PGD*, *FTL* and *TXNRD1*) were in accordance with the expression of sequencing data. *NQO1* showed no different expression in RT-qPCR , in contrast with the up-regulation observed in the sequencing data. (** $p<0.01$).

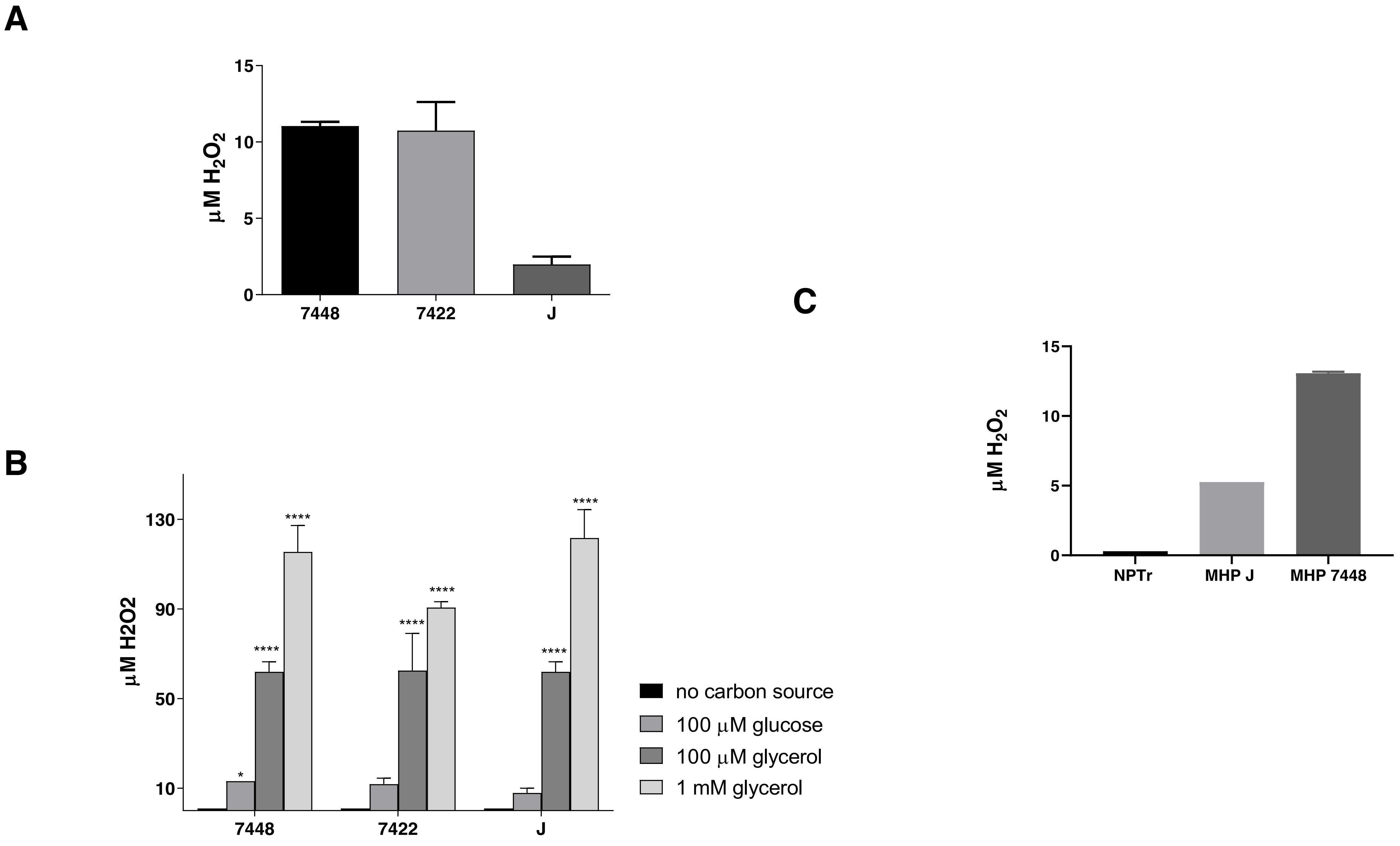

Figure S4. Production of hydrogen peroxide by different *M. hyopneumoniae* strains. A. In defined medium after bacterial growth: Hydrogen peroxide was slightly detected in growth media from the attenuated strain J. Data are presented as mean and standard deviation of three independent samples B. In the presence of different carbon sources: Pathogenic and attenuated strains of *M. hyopneumoniae* were used to test hydrogen peroxide production in incubation buffer supplemented with either glycerol or glucose after 2 h of incubation. All strains were able to produce significant amounts of the toxic product when glycerol was present. C. In the medium of NPTr cells after 24 h of infection with *M. hyopneumoniae* in the presence of glycerol. Data are represented as mean and standard deviation of four independent samples (* $p<0.05$; **** $p<0.0001$).

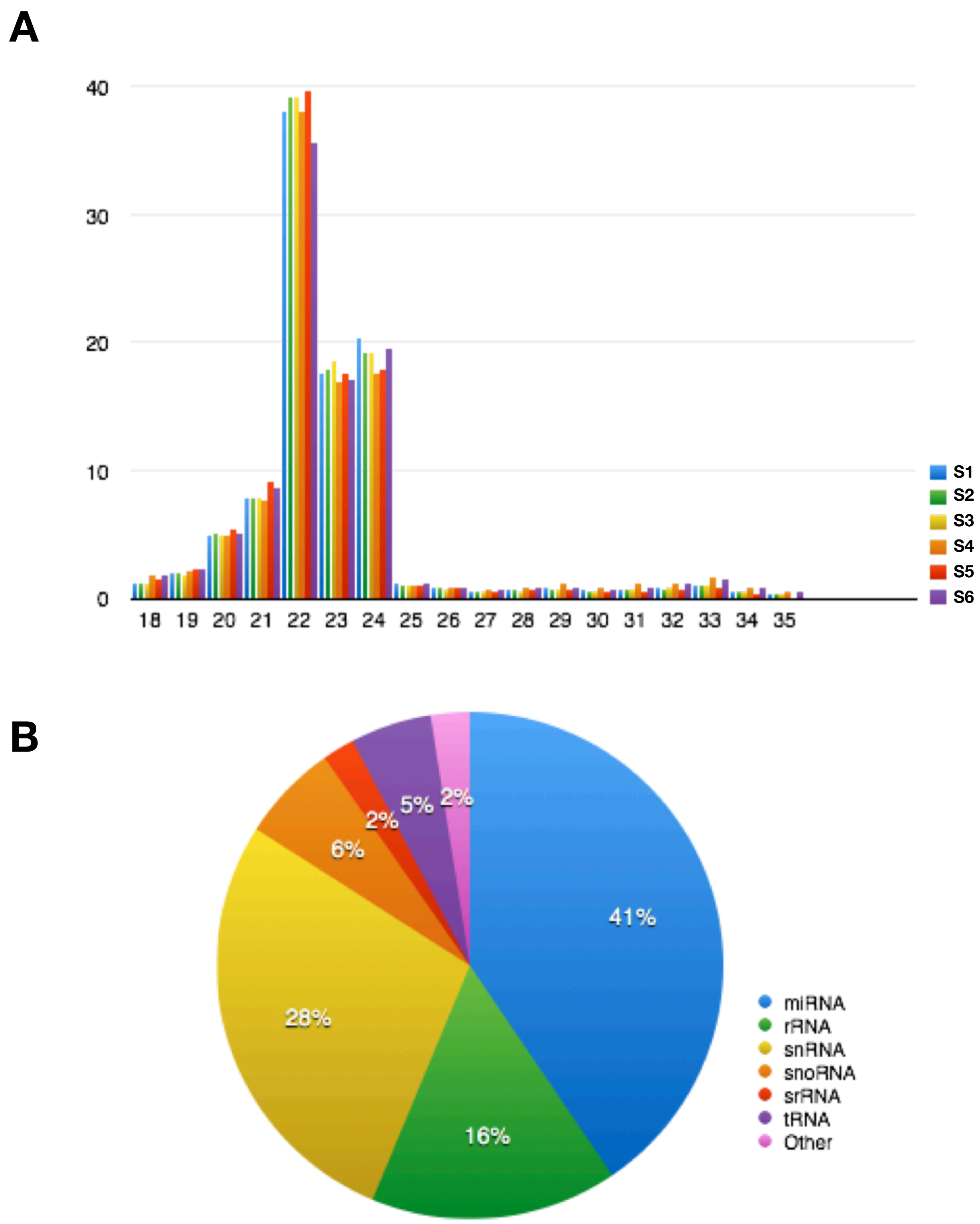

Figure S5. Distribution of intracellular sRNAs sizes and types. A. Intracellular sRNAs showed a pronounced peak at 22nt, in accordance with a typical miRNA-type size distribution curve. **B.** The distribution of reads based on homology showed that the most predominant portion of intracellular sRNAs clean reads (41%) were similar to previously described miRNAs contained in RFAM database (source: **https://rfam.xfam.org/**).

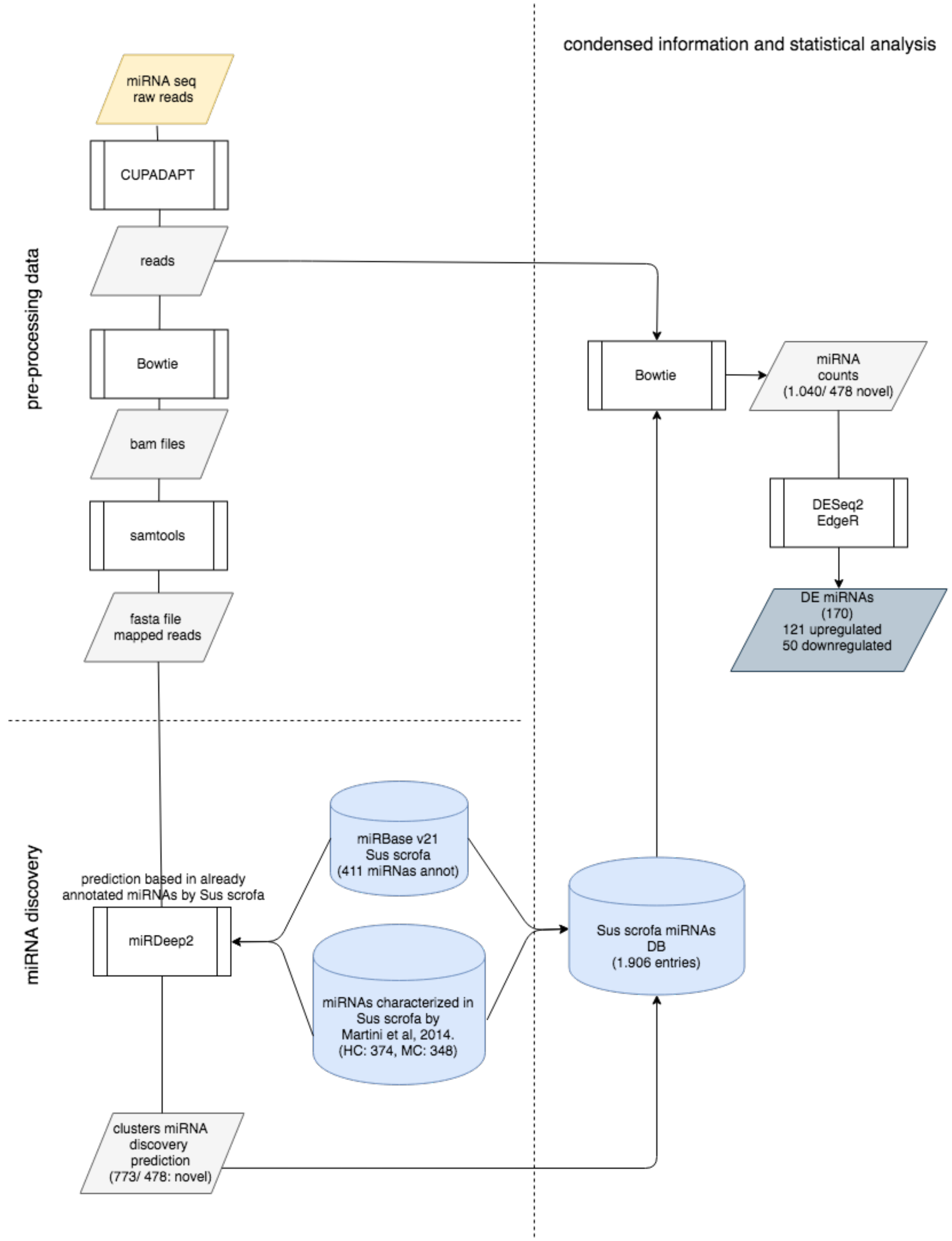

Figure S6. Complete pipeline for miRNA mapping and prediction. After filtering low quality reads and trimming adapters with CUTADAPT, clean sRNA reads were mapped against the porcine genome with Bowtie. Intracellular sRNA samples were used as input for miRDeep2 to predict miRNAs and we kept predictions with a score of at least 5. Next, we collapsed similar predictions and obtained a total of 773 clusters (773 miRNAs), of which 478 were novel miRNAs. These predictions were inclued in a porcine miRNA DB (ssc-miRNA-DB) along with the 411 annotated porcine miRNAs in version 21 of miRBase and with the 722 miRNAs characterized by Martini *et al.* (2014). Reads from all samples were mapped against this database with Bowtie and a matrix of counts was generated in order to identify DE miRNAs. Differential expression was performed with miRNAs that had at least 50 counts across all libraries. DESeq2 and EdgeR were used for intracellular and extracellular samples and GFOLD was used for exosome sequences. In total were identified 170 miRNAs DE, of which 121 were up-regulated anda 50 were down-regulated.

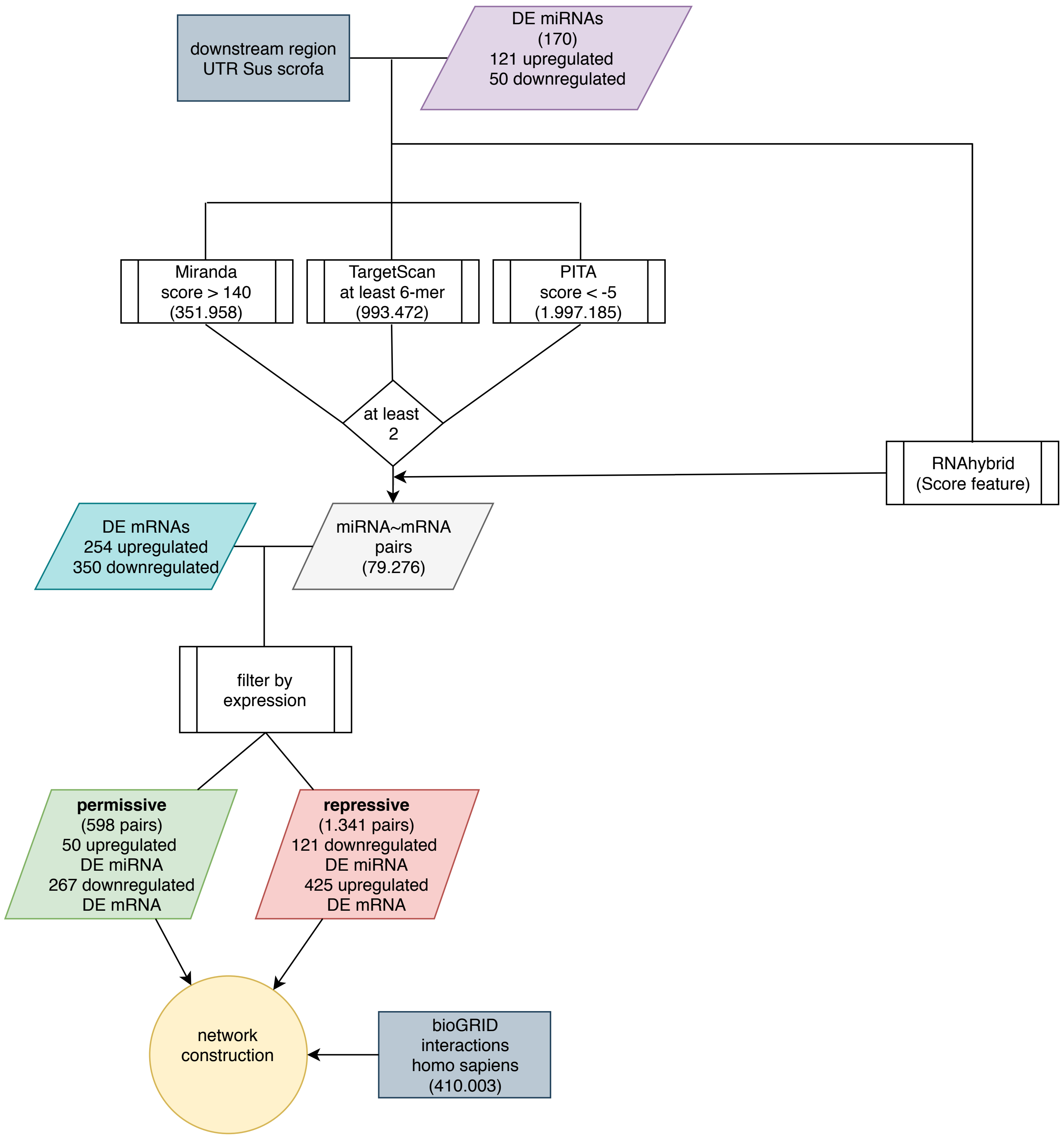

Figure S7. Complete pipeline for miRNA target prediction. DE miRNAs were used as input to detect putative interactions with the 3'UTRs of Ensembl transcripts in the porcine genome. TargetScan, miRanda and PITA were used to detect target pairs and RNAhybrid was used to validate the hybridization of a target pair. The following thresholds were used: score in miRanda > 140, DDG from PITA <-5, score in RNAhybrid <-15 and prediction in TargetScan of at least 6mers. Only targets predicted with a good score for at least two distinct tools were kept. After these, we selected from the list only the target genes that were detected as DE in this study, and subsequentially we only considered target pairs of miRNA-mRNA that had inversed fold change expression. These pairs with inversed correlation (permissive and repressive) were used for the network reconstruction in Cytoscape along with information about interactions from bioGRID.

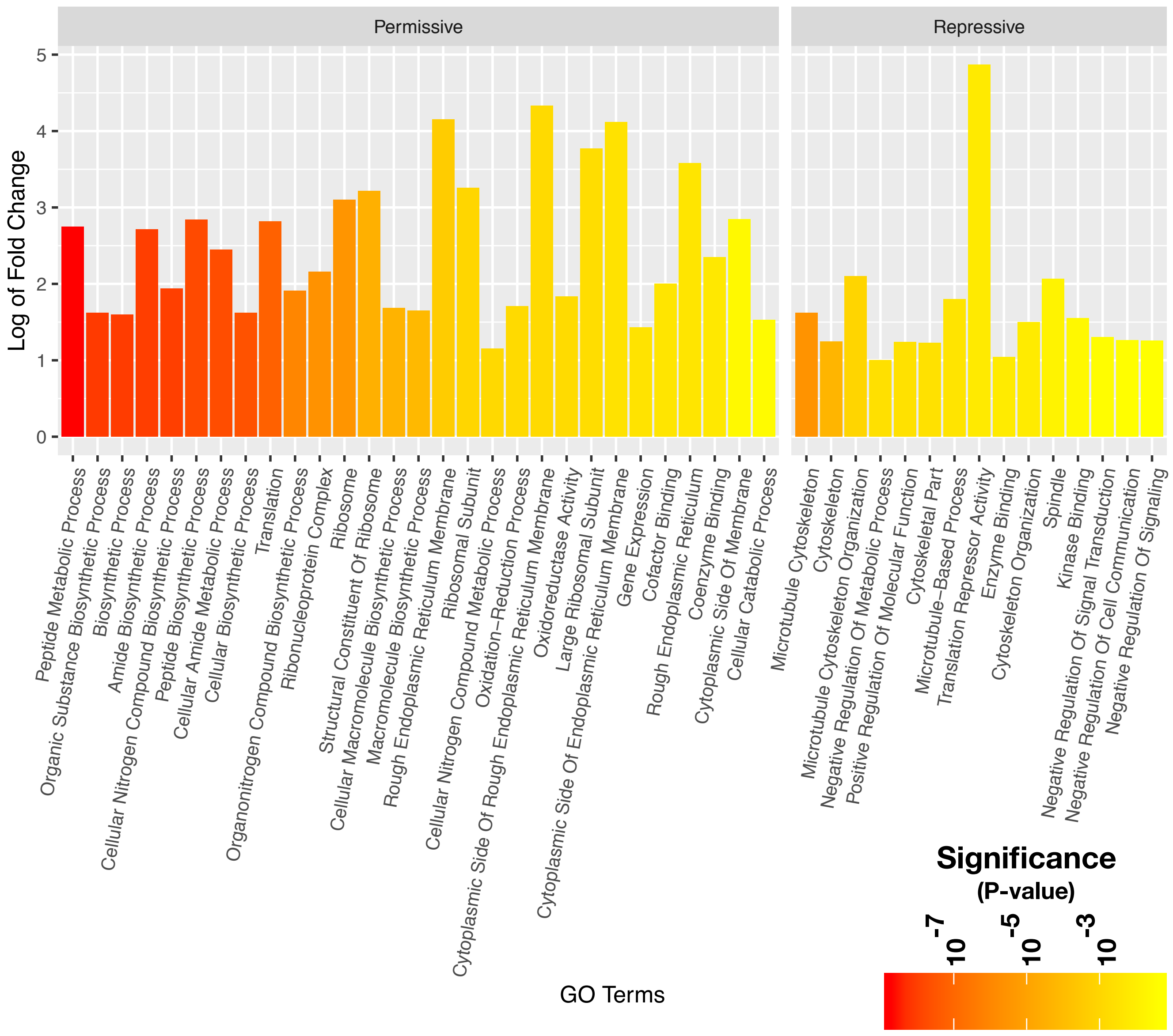

Figure S8. Complete GO enrichment analysis for miRNA targets. Target genes from permissive interactions were enriched in terms related to ribosome/translation and oxidation-reduction activity, whereas target genes from the repressive interactions were associated to cytoskeleton and cilliary function.

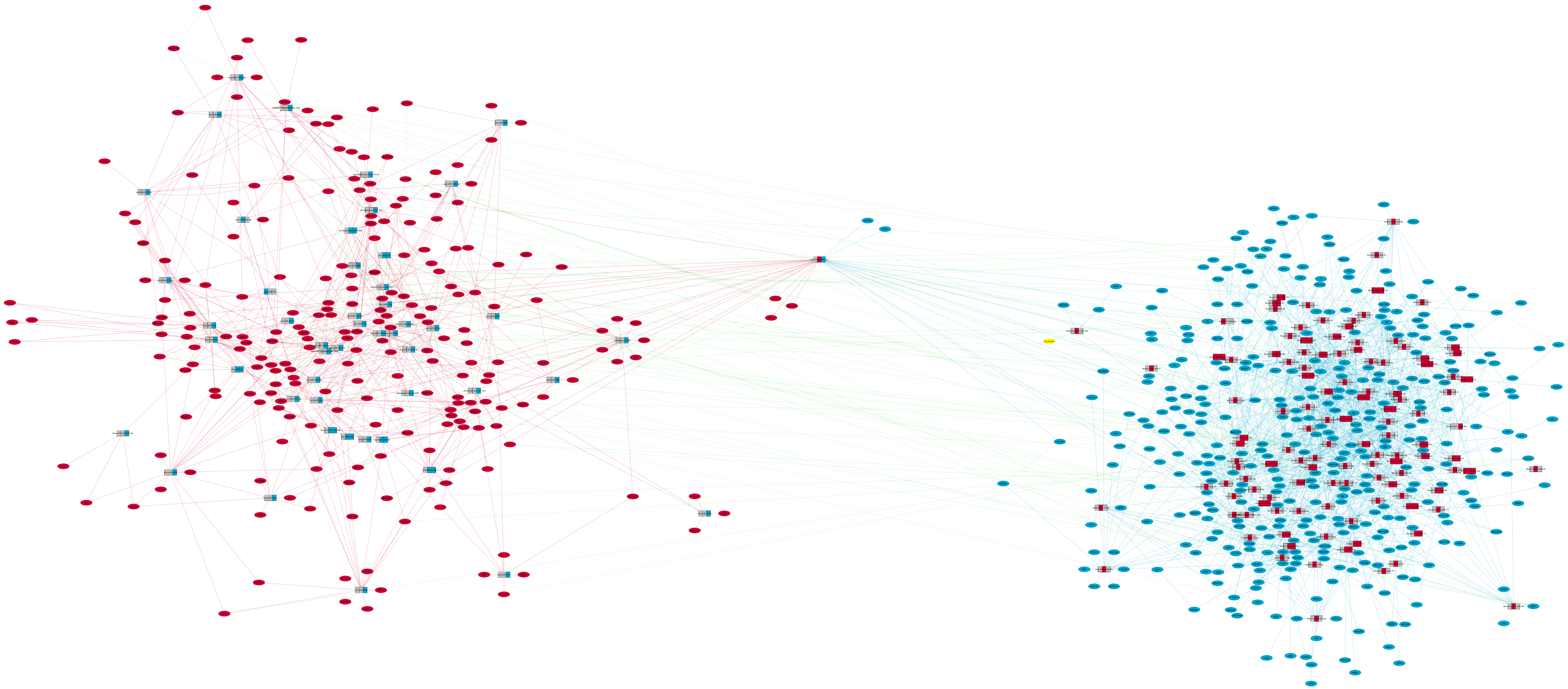

Figure S9. Complete network of permissive and repressive pairs. The complete network is also provided in .cys format (Supplementary File S1) for direct browsing and manipulation in Cytoscape software.

Permissive Targets of DE miRNAs

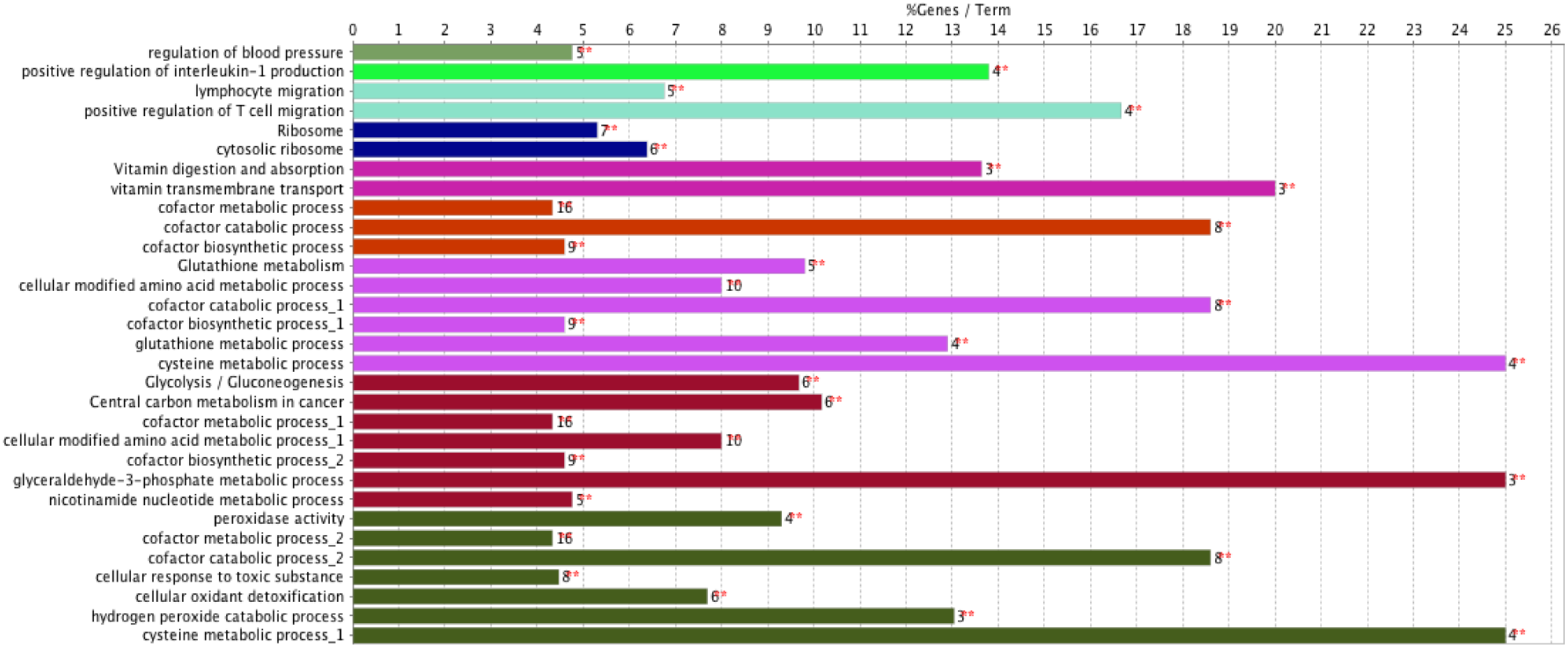

Repressive Targets of DE miRNAs

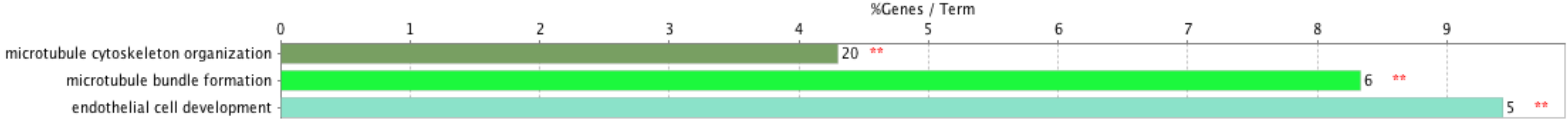

Figure S10. ClueGO analysis of the repressive and permissive pairs. In the repressive interactions we detected the enrichment of terms related to cytoskeleton, while in the permissive interactions, besides terms related to translation and oxidation-reduction activity, there was an enrichment of several processes related to immune response and inflammation.This analysis was performed with the complete list of target genes in either repressive or permissive interaction networks with a threshold for p-value of 0.01.
